# Cost-effectiveness of Ezetimibe plus statin lipid-lowering therapy: A systematic review and meta-analysis of cost-utility studies

**DOI:** 10.1371/journal.pone.0264563

**Published:** 2022-06-16

**Authors:** Akhil Sasidharan, Bhavani Shankara Bagepally, S. Sajith Kumar, Kayala Venkata Jagadeesh, Meenakumari Natarajan

**Affiliations:** 1 Health Technology Assessment Resource Centre, Indian Council of medical Research-National Institute of Epidemiology, Chennai, India; 2 Health Technology Assessment in India Secretariat, Department of Health Research, Government of India, New Delhi, India; Integral University, INDIA

## Abstract

In addition to statin therapy, Ezetimibe, a non-statin lipid-modifying agent, is increasingly used to reduce low-density lipoprotein cholesterol and atherosclerotic cardiovascular disease risk. Literature suggests the clinical effectiveness of Ezetimibe plus statin (EPS) therapy; however, primary evidence on its economic effectiveness is inconsistent. Hence, we pooled incremental net benefit to synthesise the cost-effectiveness of EPS therapy. We identified economic evaluation studies reporting outcomes of EPS therapy compared with other lipid-lowering therapeutic agents or placebo by searching PubMed, Embase, Scopus, and Tufts Cost-Effective Analysis registry. Using random-effects meta-analysis, we pooled Incremental Net Benefit (INB) in the US $ with a 95% confidence interval (CI). We used the modified economic evaluations bias checklist and GRADE quality assessment for quality appraisal. The pooled INB from twenty-one eligible studies showed that EPS therapy was significantly cost-effective compared to other lipid-lowering therapeutic agents or placebo. The pooled INB (95% CI) was $4,274 (621 to 7,927), but there was considerable heterogeneity (I^2^ = 84.21). On subgroup analysis EPS therapy is significantly cost-effective in high-income countries [$4,356 (621 to 8,092)], for primary prevention [$4,814 (2,523 to 7,106)], and for payers’ perspective [$3,255 (571 to 5,939)], and from lifetime horizon [$4,571 (746 to 8,395)]. EPS therapy is cost-effective compared to other lipid-lowering therapeutic agents or placebo in high-income countries and for primary prevention. However, there is a dearth of evidence from lower-middle-income countries and the societal perspective.

## Introduction

Cardiovascular disease (CVD) and cardiovascular events have a perpetual relationship with hyperlipidemia [1–3]. The World Health Organization (WHO) reported that an estimated 17.9 million people died from CVDs in 2019 alone, representing one-third of all global deaths [4]. For reducing cardiovascular events, statin drugs targeting 3-hydroxy-3-methyl-glutaryl-coenzyme (HMG-CoA) reductase are the most prescribed medications [5]. Statins lower the cardiovascular risk in all groups [6–8], as well as the risk of developing CVD events [9, 10]. Despite rigorous statin regimens aimed to lower the risk of cardiovascular complications, a large number of statin-treated patients fail to attain the recommended target low-density lipoprotein-cholesterol (LDL-C) levels due to statin intolerance or discontinuation of treatment due to adverse drug reactions [11, 12]. Ezetimibe is a non-statin lipid-modifying agent targeting the Niemann‐Pick C1‐like 1 intestinal cholesterol transporter protein (cholesterol absorption inhibitor) [13, 14]. It is used to achieve the desirable LDL-C levels for patients on maximally tolerated statin therapy. When added to a statin, Ezetimibe achieves a reduction in LDL-C of typically 20–25% with reduced atherosclerotic CVD (ASCVD) risk [15, 16]. Hence, the 2018 Cholesterol guidelines warrant using Ezetimibe in individuals with a high ASCVD risk despite receiving optimal statin medication [7]. Studies showed that Ezetimibe reduced LDL-C at levels consistent with Cholesterol Treatment Trialists’ (CTT) Collaboration estimates, giving CTTs’ extrapolation beyond their initial analysis legitimacy and validity [15, 17]. Many professional organisations have recently issued guidelines recommending the use of non-statin medications in clinical practice, considering their usefulness [3, 18–20].

Ezetimibe co-administration with statins has resulted in fewer very-high-risk and extremely high-risk patients eligible for other lipid-lowering agents [21]. A favourable tolerability profile, ease of use, and affordability make Ezetimibe a better option than PCSK9i [22]. Furthermore, a recent meta-analysis of cost-utility studies (CUA) showed PCSK9i to be not cost-effective compared to other lipid-lowering therapeutic agents in high-income countries (HICs) [23, 24]. As a result, Ezetimibe can be used as a next- cholesterol medication. However, its use and the extent to which it meets unmet requirements are limited. The availability of Ezetimibe as a generic drug in several countries [25] can act as a positive indicator and increase overall access to this medication.

Further, the evidence on the cost-effectiveness of this therapy is also inconsistent, as few studies report it is cost-effective [26, 27]. In contrast, other studies report that combination therapy is not cost-effective [20, 28] compared to statin therapy. Hence to provide syntheised evidence, we systematically reviewed the evidence on the cost-effectiveness and quantitatively estimated the pooled incremental net benefit (INB) of ezetimibe therapy.

## Materials and methods

The study protocol was pre-registered with PROSPERO, CRD42021248531, and the study was conducted according to the Preferred Reporting Items for Systematic Reviews and Meta-analyses (PRISMA) [29].

### Data sources, eligibility criteria, screening, and search strategy

We searched PubMed, Embase, Scopus, and the Tufts Medical Centers’ cost-effective analysis (CEA) registry [30] for studies published from inception to 26 April 2021 (S1 Appendix in S1 File), adhering to the PRISMA guideline. We followed the PICO approach (Population, Intervention, Comparator, Outcome) to construct the search terms. Published Cost-Utility Analysis (CUAs) were eligible if they met all the following inclusion criteria. Adult subjects (age above 18 years) with risk of or established CVD requiring lipid-lowering therapy and treated with Ezetimibe compared to other lipid-lowering therapeutic agents such as statins or PCSK9i, or with placebo/no treatment. We included studies reporting economic outcomes in incremental cost-effectiveness ratios (ICER) per quality-adjusted life years (QALYs) or INB. Studies with effectiveness measured other than in quality-adjusted life years (QALY), reviews, letters, editorials, abstracts, books, reports, grey literature, and methodological articles were excluded. The detailed search terms are reported in S1 Appendix in S1 File.

### Selection of studies

**Fig 1:**
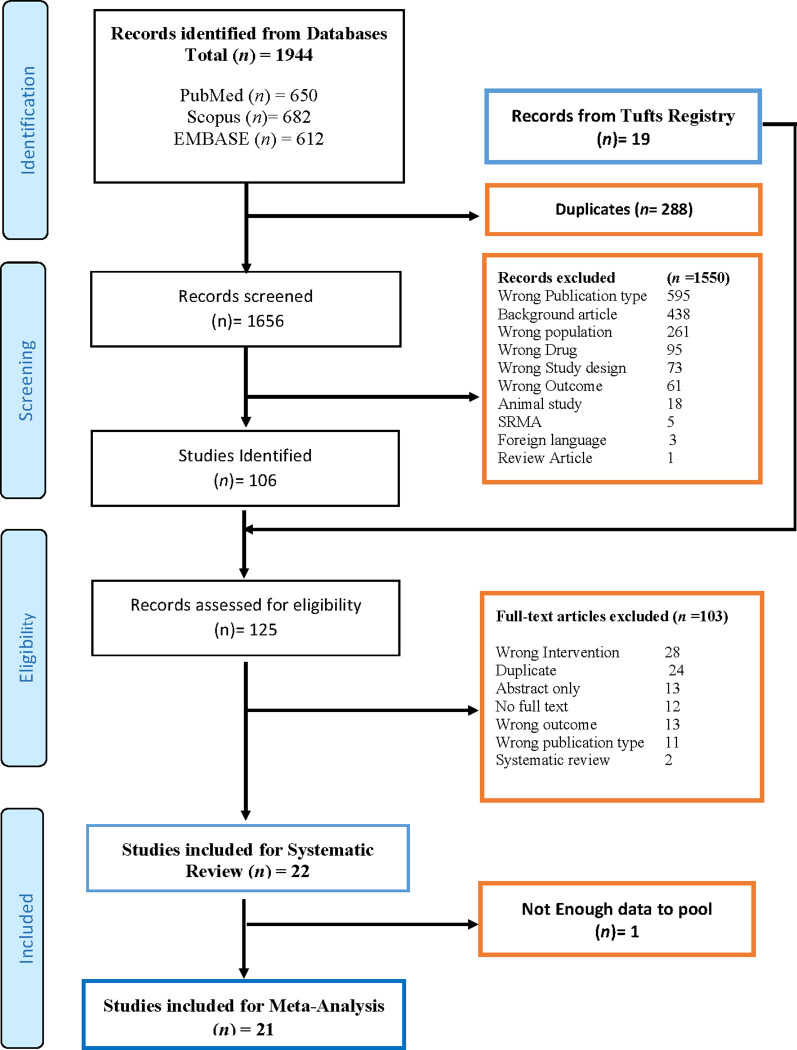
PRISMA flow chart.

We identified a total of 1,944 studies after systematically searching multiple peer-reviewed repositories. All English language studies that met the eligibility criteria listed from the electronic database search were screened independently for titles and abstracts by two independent reviewers (BSB and AS) for their potential inclusion using the Rayyan-web application [31]. Reviewers (AS, KVJ, and MK) independently reviewed the full text of the finalized 125 studies after the title and abstract screening and deduplication in detail. The independent assessors’ mutual agreement with another reviewer (BSB) produced the final list of studies meeting the inclusion and exclusion criteria (n = 22), and data were extracted from the selected studies. The PRISMA flow chart of the screening process is appended as Fig 1.

### Data extraction

Using a data extraction template adapted for the outcomes of interest, we extracted the following data from eligible studies: author, year, country of setting, study/patient characteristics, intervention, comparator, and the general characterization of the model, which included model type, perspective, time horizons, discount rate, and currency year. We extracted economic parameters such as costs (C), incremental costs (ΔC), clinical effectiveness (E), its incremental effectiveness (ΔE), ICERs, INB values, and their measures of dispersion [i.e., standard deviation (SD), standard error (SE), or 95% confidence interval (CI), willingness to pay (WTP), and threshold (K). From the cost-effective (CE) plane graph, we extracted ΔC and ΔE values using Web-Plot-Digitaliser [32].

### The outcome of interest

In the meta-analysis, we estimated the pooled INB, defined as pooled INB = K*ΔE-ΔC, where K was the WTP threshold, ΔC-incremental cost (i.e., the difference in costs between intervention and comparator), ΔE-incremental effectiveness (i.e., the difference in effectiveness between intervention and comparator). A positive INB favours intervention, i.e., intervention is cost-effective, whereas a negative INB favours the comparator, i.e., intervention is not cost-effective. INB is used instead of ICER as the effect measure because of the inherent limitations of ICER and the ambiguity in interpreting them [33–35].

### Data preparation and statistical analysis

We followed the data preparation method and analysis described and used elsewhere [23, 33, 36–38]. To calculate the INB and its variance, mean values along with dispersions (SD, SE, and 95% CI) of ΔC and ΔE are required. However, economic studies reported different parameters; therefore, we designed five scenarios to deal with the data available from primary studies (S2 Appendix in S1 File). Using the data reported in the primary research publications and following the approach detailed in Bagepally et al. [39], we calculated the INB and its variances for each intervention comparator duo. If a cost-effective (CE) plane graph was not provided, covariance was estimated for 1000 Monte-Carlo simulations from the extracted ΔC and ΔE values for studies included in scenario 3 (S2 Appendix in S1 File).

Included studies reported in different currencies from different time points (years). To compare INB in a common currency, all monetary units, except for the non–GDP-based threshold, were adjusted for inflation using the consumer price index (CPI) and converted to purchasing power parities (PPP)-adjusted US dollar (US $) for the year 2021, as detailed in Appendix II in S1 File. Following the data preparation, INBs were pooled across studies stratified by income classification as low-income (LIC), lower-middle (LMIC), upper-middle (UMIC), and high-income (HIC) countries as per the World Bank classification [40]. Meta-analysis was applied to pool the INBs using random-effects model based on the DerSimonian and Laird method. I^2^ statistics were used to assess heterogeneity, I^2^ > 50% was considered substantial heterogeneity, and Cochrane Q p-value < 0.05 was taken as a cut-off for significant heterogeneity. We did subgroup analysis wherever appropriate to explore the source of heterogeneity and provide subgroup-specific pooled INBs. Subsequently, we assessed the publication bias using funnel plots and Eggers’ test. Furthermore, we explored the sources of asymmetry using contour-enhanced funnel plots. All data were prepared using Microsoft Excel version 2019 [41] and analyzed using Stata software version 16 [42].

### Risk of bias assessment and quality assessment

Reporting quality was assessed independently by the reviewers using the modified economic evaluation bias (ECOBIAS) checklist [43]. It considers both overall biases (11 items) and model-specific biases, including structure (4 items), data (6 items), and internal consistency (1 item). Each item was rated as applicable, partially applicable, unclear, no, or not applicable (S1 Fig in S1 File). GRADE (Grading of Recommendation, Assessment, Development, and Evaluation) was used to assess the quality of evidence and grade recommendations [44, 45]. We graded the evidence for the cost-effectiveness of Ezetimibe plus statin (EPS) therapy compared to other lipid-lowering therapeutic agents or a placebo. The certainty of the evidence was rated for EPS therapy in HICs, for primary prevention, from a payer’s perspective, and over a lifetime horizon. Additionally, the certainty of the evidence was rated for the cost-effectiveness of EPS therapy versus statin monotherapy, for EPS therapy versus statin monotherapy for primary prevention from the payer’s perspective as well. This assessment was based on the risk of bias, inconsistency, indirectness, imprecision, publication bias, and other considerations. The quality of the evidence was classified as high, moderate, low, or very low [44, 45]. We excluded study design and sample size considerations because the included cost-utility studies are model-based.

## Results

### General characteristics of the included studies

We retrieved and included twenty-two potentially relevant articles [20, 26–28, 46–63] for systematic review, of which 21 studies were eligible for meta-analysis [20, 26–28, 46–48, 50–63] (Fig 1). One study from the UK, Ara et al. [49], was not included for meta-analysis due to incomplete data. The general characteristics of the included articles are summarised in Table 1.

**Table 1:** Characteristics of identified studies for systematic review and meta-analysis.

Author_ year	Country	Setting	Perspective	Target population	Time Horizon	Discount Rate (%)	Reference year	Intervention	Comparator	Prevention	Original reported findings
Kohli_2006 [26]	Canada	Risk Group	Healthcare	CAD & non-fatal CAD (angina or acute MI)	Lifetime	5	2002	Ezetimibe+ Statin	Statin	PP+SP	CE
Ara_2008^1^ [47]	UK	Country	Healthcare	Primary Hypercholesterolemia	Lifetime	3.5	2006	Ezetimibe+ Statin	No Treatment	PP	CE
Ara_2008^2^ [48]	UK	Risk Group	Healthcare	Primary Hypercholesterolemia	Lifetime	3.5	2006	Ezetimibe+ Statin	Statin	PP	CE
Ara_2008^3^ [49]*	UK	Risk Group	Healthcare	ACS	Lifetime	3.5	2006	Ezetimibe+ Statin	Statin		CE
Reckless_2010 [50]	USA	Risk group	Payer	Non-fatal CHD with or without DM	Lifetime	3.5	2004	Ezetimibe+ Statin	Statin	SP	CE
Soini_2010 [51]	Finland	Risk group	Societal	Primary Hypercholesterolemia	Lifetime	3	2007	Ezetimibe+ Statin	Statin	SP	CE
Nooten_2011 [52]	Netherland	Country	Societal	High CVD risk, history of CHD and/or DM	Lifetime	4	2008	Ezetimibe+ Statin	Statin	PP	CE
Laires_2015 [27]	Portugal	Country	Payer	CKD but without known CHD	Lifetime	5	2015	Ezetimibe+ Statin	Statin	PP	CE
Mihaylova_2016 [53]	UK	Country	Healthcare	HeterozygousFH/ Preexisting ASCVD	Non-Lifetime	3.5	2015	Ezetimibe+ Statin	No Treatment	PP	Not CE
Kazi_2016 [46]	USA	Country	Healthcare	HeterozygousFH/ Preexisting ASCVD	Lifetime	3	2015	Ezetimibe+ Statin	Statin	SP	CE
				ASCVD	Lifetime	3	2015	Ezetimibe+ Statin	PCSK9i+ Statin	SP	CE
Kazi_2017 [55]	USA	Risk Group	Healthcare	ACS	Lifetime	3	2017	Ezetimibe+ Statin	Statin	SP	CE
Almalki_2017 [57]	Saudi Arabia	Country	Healthcare	CVD history (both CHD and stroke).	Lifetime	3	2016	Ezetimibe+ Statin	Statin	SP	CE
Davies_2017 [54]	USA	Country	Payer	CAD & non-fatal CAD (angina or acute MI)	Lifetime	3	2013	Ezetimibe+ Statin	Statin	PP+SP	CE
Stam-Slob_2017 [56]	Netherland	Risk group	Healthcare	stable CAD	Lifetime	4	2014	Ezetimibe+ Statin	Statin	SP	NA
Korman_2018 [63]	Norway	Country	Healthcare	Hypercholesterolemia, DM, Statin Intolerant	Lifetime	4	2015	Ezetimibe+ Statin	Statin	PP+SP	CE
Stam-Slob_2018 [58]	Netherland	Risk group	Healthcare	FH without a history of vascular disease, stable vascular disease, DN	Lifetime	3	2014	Ezetimibe+ Statin	PCSK9i+ Ezetimibe+ Statin	SP	NA
Kongpakwattana_2019 [28]	Thailand	Country	Societal	Existing CVD, comprising MI and stroke	Lifetime	3	2018	Ezetimibe+ Statin	Statin	SP	Not CE
			Healthcare	Existing CVD, comprising MI and stroke	Lifetime	3	2018	Ezetimibe+ Statin	Statin	SP	Not CE
Kazi_2019 [62]	USA	Country	Healthcare	ACS	Lifetime	3	2018	Ezetimibe+ Statin	Statin	SP	CE
				ACS	Lifetime	3	2018	Ezetimibe+ Statin	PCSK9i+ Statin	SP	CE
Schlackow_2019 [60]	USA	Risk group	Healthcare	Non dialysis patients with CKD	Lifetime	3	2015	Ezetimibe+ Statin	Statin	PP	CE
	UK	Risk group	Healthcare	Non dialysis patients with CKD	Lifetime	3.5	2015	Ezetimibe+ Statin	Statin	PP	CE
Dressel_2019 [59]	Germany	Country		Stable CAD	Lifetime	3	2018	Ezetimibe+ Statin	PCSK9i+ Ezetimibe+ Statin	SP	Not CE
Landmesser_2020 [20]	Sweden	Risk group	Payer	Recent MI /MI with a second event/MI with a risk factor	Lifetime	3	2019	Ezetimibe+ Statin	PCSK9i+ Ezetimibe+ Statin	SP	Not CE
Han Yang_2020 [61]	China	country	Healthcare	Newly diagnosed with CVD	Non-Lifetime	3	2017	Ezetimibe+ Statin	Statin	SP	CE

PP-primary prevention, SP-secondary prevention, PP+SP- both primary and secondary prevention, CE- cost effective

*Not included in meta-analysis

Included studies reported 25 comparisons; EPS therapy versus statin monotherapy(n = 18) [26–28, 46, 48, 50–52, 54–57, 60–63], EPS therapy versus PCSK9i plus Ezetimibe and statins(n = 3) [20, 58, 59], EPS therapy versus PCSK9i with statin therapy(n = 2) [46, 62] and EPS therapy versus no treatment or placebo(n = 2) [47, 53]. All studies were set in HICs, except two, based on UMICs [28, 61]. Most of the studies (n = 20) analysed a lifetime horizon, except two studies [53, 61]. Fourteen studies reported from a healthcare perspective [26, 28, 46–48, 53, 55–58, 60–62], four from payer’s perspective [20, 27, 50, 54], and three studies from a societal perspective [28, 51, 52]. All studies used Markov models with a cycle length of one year, except for an alongside trial [53]. The named models included were Cardio Vascular Disease Policy Model (CVDPM) (n = 3) [46, 55, 62] and the COOK model (n = 2) [26, 54]. All studies used discounting for cost and outcomes. All of the included studies reported direct medical costs; besides, Kongpakwattana et al. reported direct non-medical costs [28], and Landmesser et al. reported indirect costs [20]. All the studies reported using country-specific thresholds as WTP except one study [61] that used GDP-based WTP.

In the meta-analysis, due to the differences in reported outcomes among different studies, the INB variance from the most comparable studies was utilised for calculations under scenario five (S2 Appendix in S1 File). The INB variance of Van Nooten et al. [52] was used for three studies [20, 51, 56], and the INB variance of Schlackow et al. [60] was used for six studies [26, 27, 47, 48, 53, 57], respectively. Among the 21 studies evaluating the cost-effectiveness of Ezetimibe versus other lipid-lowering therapeutic agents or placebo, seven were set in Europe [20, 27, 51, 52, 58, 59, 63]; five were set in the US [46, 50, 54, 55, 60, 62], three in the UK [48, 49, 53] and one studies each from Canada [26], China [61], Saudi Arabia [57] and Thailand [28]. Schlackow et al. reported from the health system perspective of both the US and UK [60]. In all studies, except Kongpakwattana et al. [28] and Almalki et al. [57] mean plasma LDL-C levels less than70 mg/dL reflected current ezetimibe dosing recommendations. Except for two [50, 51] that included fatal and non-fatal stroke as well, all studies included fatal and non-fatal coronary heart diseases in their model. Unstable angina was modelled in three studies [50, 51, 57] in which Almalki et al. [57] considered coronary revascularisation also. Even though three studies [46, 55, 62] modelled adverse events as consequences, only Yang et al. [61] included costs due to adverse events. Six studies profiled the model population after local registries and databases [46, 51, 54, 55, 62, 63], three studies [50, 57, 61] from clinical trials, only Kongpakwattana et al. used data from a meta-analysis of RCTs [28] (Table 1).

All three studies from payers’ perspective [27, 50, 54], and seven studies out of nine from the healthcare perspective [26, 28, 48, 55–57, 60–62] and two out of three studies from the societal perspective [51, 52] reported EPS therapy to be cost-effective compared with statin monotherapy. From the healthcare perspective, all two studies [46, 62] considered EPS therapy to be cost-effective compared to PCSK9i with statin therapy, but only one study [58] out of three, reported EPS therapy to be cost-effective compared with PCSK9i along with Ezetimibe and statin. The alongside trial [53], which compared EPS therapy and placebo, reported that the intervention was not cost-effective. In contrast, a model-based study [47] that compared EPS therapy versus no treatment reported the intervention as cost-effective. In addition, according to the WTP threshold of Thailand, Kongpakwattana et al. [28] reported Ezetimibe as not cost-effective from healthcare or societal perspective. Three studies did not report any sensitivity analyses [50, 55, 63] while the remaining studies [20, 26–28, 46–48, 51, 52, 54, 56–58, 61, 62] included deterministic as well as probabilistic sensitivity analyses. Additionally, eight studies [28, 46–48, 52, 56, 57, 62] reported scenario and threshold analyses, and six studies [26, 51, 53, 54, 58, 62] also included sub-group analyses.

### Quality appraisal

#### Risk of bias assessment

The risk of bias among the identified studies was analysed using the ECOBIAS checklist [64]. The ECOBIAS checklist shows that almost 91 percent of the studies chose the best current practice as a comparator, and all the comparators are adequately described. The details of the data used in the studies are transparent. All studies provided sufficient detail on the costs, effectiveness, discount rates, and have acknowledged the sources of funding. Additionally, model selection bias was negligible. Similarly. bias related to time horizon was also low since majority of the studies employed a lifetime horizon. Limited scope bias is highly probable in almost all studies, also internal consistency related to mathematical logic was not evident (S1 Fig in S1 File).

#### Publication bias

The funnel plot showed asymmetry. The studies were distributed along with the mean effect size of the funnel plot. The Egger’s test with a higher p-value (p = 0.860) indicates no significant variability among the studies and no publication bias. However, the absence of studies in the area of significance on the contour enhanced funnel plot suggests the possibility of publication bias due to factors other than non-reporting bias (S2 Fig in S1 File). Due to high heterogeneity, it would be difficult to distinguish between publication bias and other causes.

### Ezetimibe compared with other lipid-lowering therapeutic agents or placebo

**Fig 2:**
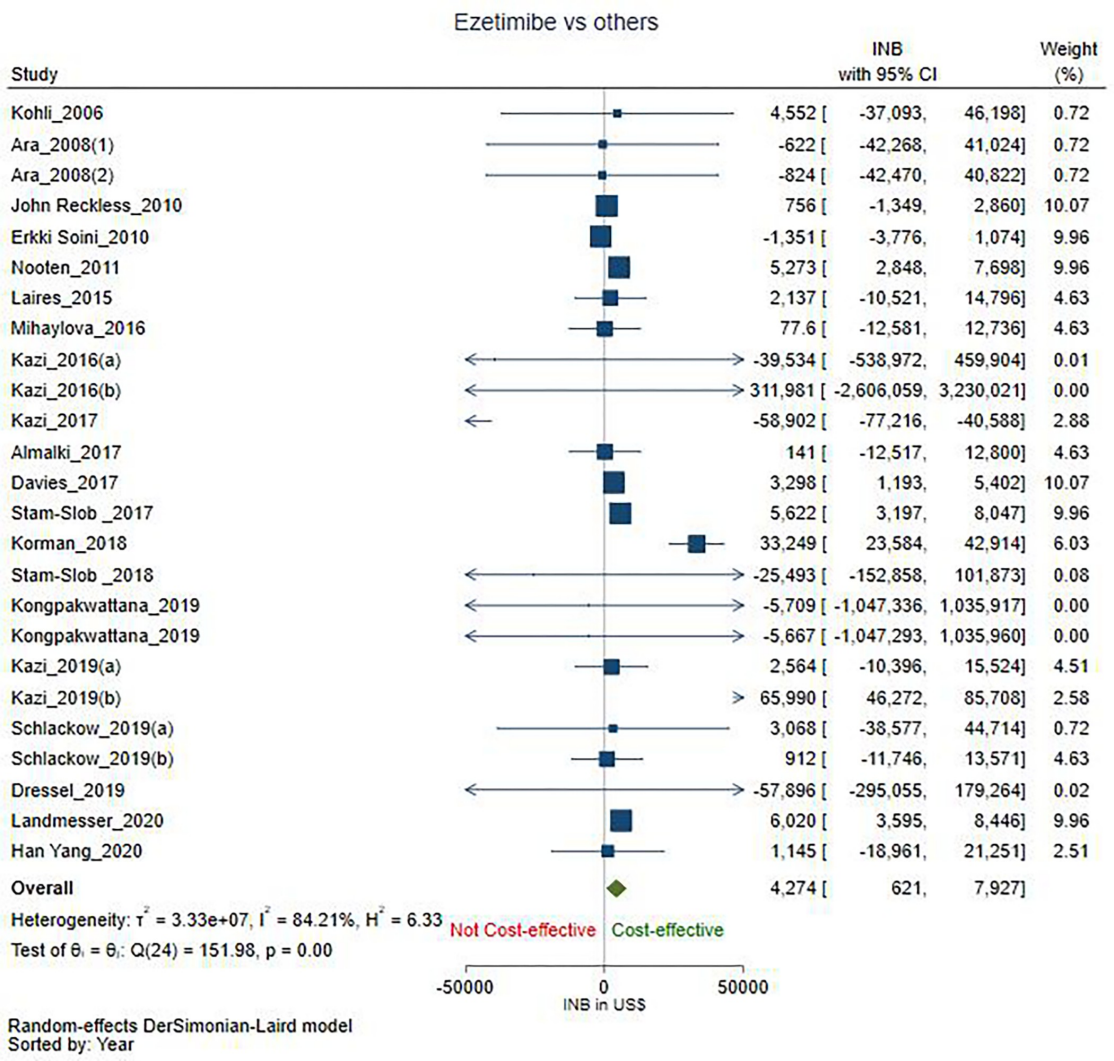
Forest plot.

The pooled INB (INBp) with 95% CI, $4,274 (621 to 7,927) showed EPS therapy is significantly cost-effective compared with other lipid-lowering therapeutic agents or placebo. The INBp calculated from 25 comparisons [20, 26–28, 46–48, 50–63] revealed considerable heterogeneity (I^2^ = 84.21) (Fig 2).

#### Subgroup analysis

We conducted subgroup analyses to explore the heterogeneity between studies and provide subgroup specific pooled INBs. Subgroup analysis based on treatment comparisons showed that EPS therapy is significantly cost-effective compared with PCSK9i plus statin therapy (n = 2) [46, 62] with INBp $66,001 (46,284 to 85,718). Also, EPS therapy is significantly cost-effective compared to PCSK9i plus EPS therapy (n = 3) [20, 58, 59] with INBp $6,002 (3,578 to 8,427). There was no heterogeneity (I^2^ = 0.0) in either of the subgroups. However, EPS therapy is not significantly cost-effective compared to statin monotherapy (n = 18) [26–28, 46, 48, 50, 51, 54–57, 60–63] with INBp $2,558 (-1,249 to 6,364) and considerable heterogeneity (I^2^ = 84.02). Likewise, EPS therapy is not significantly cost-effective compared to no treatment or placebo (n = 2) [47, 53] with INBp $18.4 (-12,093 to 12,130) and no heterogeneity (I^2^ = 0.0) (S3 Fig in S1 File).

On subgroup analysis with the income status of the countries, the pooled INBs from HICs (n = 22) [20, 26, 27, 46–48, 50–60, 62, 63] showed that EPS therapy is cost-effective compared with other lipid-lowering therapeutic agents or placebo with an INBp of $4,356 (621 to 8,092) with considerable heterogeneity (I^2^ = 86.18). In contrast, EPS therapy is not significantly cost-effective for the UMICs [28, 61] with an INBp of $1,140 (-18,959 to 21,239) and no heterogeneity (I^2^ = 0.0) (S4 Fig in S1 File).

On subgroup analysis with study perspective, the pooled INBs among studies from payers’ perspective (n = 4) [20, 27, 50, 54] showed that EPS therapy is significantly cost-effective compared with other lipid-lowering therapeutic agents or placebo with an INBp of $3,255 (571 to 5,939) but with substantial heterogeneity (I^2^ = 71.14). However, EPS therapy is not significantly cost-effective from a healthcare perspective (n = 17) [26, 28, 46–48, 53, 55–58, 60–63] with INBp $4,734 (-6,769 to 16,238) and considerable heterogeneity (I^2^ = 86.41) or from a societal perspective (n = 3) [28, 51, 52] with INBp $1,961 (-4,300 to 8,222) and considerable heterogeneity (I^2^ = 86.04) (S5 Fig in S1 File).

On subgroup analysis based on the time horizon of the study (n = 23) [20, 26–28, 46–48, 50–52, 54–60, 62, 63], EPS therapy is significantly cost-effective compared with other lipid-lowering therapeutic agents or placebo with an INBp of $4,571 (746 to 8,395) but with substantial heterogeneity (I^2^ = 85.50). However, among studies with a non-lifetime horizon, EPS therapy is not significantly cost-effective with an INBp of $381 (-10,332 to 11,093) [53, 61] and no heterogeneity (I^2^ = 0.0) (S6 Fig in S1 File).

Subgroup analysis based on discount rates of 3% (n = 15) [20, 28, 46, 51, 54, 55, 57–60, 62] with INBp $1,879 (-4,547 to 8,305), 3.5% (n = 5) [48–50, 53, 60] with INBp $735 (-1,309 to 2,778) and 5% (n = 2) [26, 27] with INBp $2,342 (-9,770 to 14,453) showed EPS therapy is not significantly cost effective compared to other lipid-lowering therapeutic agents or placebo. The 3% discount rate subgroup had considerable heterogeneity (I^2^ = 86.27), but the 3.5% and 5% discount rates subgroup had no heterogeneity (I^2^ = 0.0). However, with a discount rate of 4% (n = 3) [52, 56, 63], EPS therapy is significantly cost-effective with an INBp of $12,254 (4,448 to 20,060) and considerable heterogeneity (I^2^ = 93.52) (S7 Fig in S1 File).

EPS therapy is significantly cost-effective for primary prevention (n = 7) [27, 48, 49, 52, 53, 60] with an INBp of $4,814 (2,523 to 7,106) and no heterogeneity (I^2^ = 0.0). However, the pooled INBs showed that EPS therapy is not significantly cost-effective for secondary prevention (n = 15) [20, 28, 46, 50, 51, 55–59, 61, 62] with an INBp of $2,088 (-3,282 to 7,457) and considerable heterogeneity (I^2^ = 87.32). Similarly, for primary and secondary prevention together (n = 3) [26, 54, 63] INBp $15,257 (-10,035 to 40,550) with considerable heterogeneity (I^2^ = 94.32) showed EPS therapy is not significantly cost-effective (S8 Fig in S1 File).

The thresholds used in the comparisons ranged from PPP adjusted $13,218 to $2,05,198. Based on the median threshold of $50,000, the mean INBp among both the subgroups, viz., thresholds of >50,000 $ (n = 13) [20, 27, 46, 48, 55, 56, 58–60, 62, 63] with INBp $7,398 (-1,657 to 16,452) and considerable heterogeneity (I^2^ = 89.62) and thresholds of <50,000 $ (n = 12) [26, 28, 47, 50–54, 57, 60, 61], INBp $1,886 (-2 to 3,775) with moderate heterogeneity (I^2^ = 36.66) showed EPS therapy is not cost effective compared with other lipid-lowering therapeutic agents or placebo, but without statistical significance (S9 Fig in S1 File).

On subgroup analysis based on scenario, the mean INBp showed EPS therapy is not significantly cost effective compared with other lipid-lowering therapeutic agents or placebo for scenarios two (n = 6) [46, 55, 59, 62] with an INBp of $-522 (-59,813 to 58,769) and considerable heterogeneity (I^2^ = 93.99). Under scenarios three (n = 3) [60, 63] with INBp $14,398 (-12,225 to 41,020) and with considerable heterogeneity (I^2^ = 87.92) and four (n = 5) [28, 50, 52, 58, 61] with INBp $2,876 (-568 to 6,320) with moderate heterogeneity (I^2^ = 48.81), EPS therapy was not significantly cost effective compared with other lipid-lowering therapeutic agents or placebo. However, under scenario five (n = 11) [20, 26–28, 47, 48, 51, 53, 54, 56, 57], EPS therapy was significantly cost effective with INBs of $3,102 (599 to 5,606) and substantial heterogeneity (I^2^ = 56.67) (S10 Fig in S1 File).

Further subgroup analysis was conducted to explore the heterogeneity observed when EPS therapy was compared with statin monotherapy. We found that compared with statin monotherapy, EPS therapy is significantly cost-effective for primary prevention (n = 5) [27, 48, 52, 60] with an INBp $4,992 (2,659 to 7,326) and no heterogeneity (I^2^ = 0.0) (S11 Fig in S1 File). Also, from a payers’ perspective (n = 3) [27, 50, 54] with INBp $2,029 (72 to 3,987) and less heterogeneity (I^2^ = 28.65) EPS therapy is significantly cost-effective compared with statin monotherapy (S12 Fig in S1 File). However, EPS therapy was cost-effective but without statistical significance among the subgroups of HICs (n = 15) with INBp $2,574 (-1,341 to 6,488) and with substantial heterogeneity (I^2^ = 86.83), UMICs (n = 3) with INBp $1,140 (-18,959 to 21,239) with no heterogeneity (I^2^ = 0.0) (S13 Fig in S1 File). Similar results were also observed in healthcare perspective (n = 12) [26, 28, 46, 49, 55–57, 60–63] with INBp $-323 (-12,568 to 11,922) and substantial heterogeneity (I^2^ = 86.40), societal perspective (n = 3) [28, 51, 52] with INBp $1,961 (-4,300 to 8,222) with substantial heterogeneity (I^2^ = 86.04), primary and secondary prevention together (n = 3) [26, 54, 63] with INBp $15,257 (-10,035 to 40,550) with substantial heterogeneity (I^2^ = 94.32) and lifetime horizon (n = 17) [26–28, 46, 48, 50, 51, 54–57, 60, 62, 63] with INBp $2,589 (-1,293 to 6,471) with substantial heterogeneity (I^2^ = 84.95) (S14 Fig in S1 File). For secondary prevention (n = 10) [28, 46, 50, 51, 55–57, 61, 62] INBp $-2,303 (-7,728 to 3,123) showed that EPS therapy is not cost-effective compared to statin monotherapy but without statistical significance and substantial heterogeneity (I^2^ = 84.65).

### Certainty of evidence

The GRADE quality assessment revealed low confidence in the overall evidence of the cost-effectiveness of EPS therapy when compared with other lipid-lowering therapeutic agents or placebo. We found low confidence in results for HICs and for primary prevention. However, the confidence of results from a payer perspective and lifetime horizon is very low. Considering EPS therapy compared with statin monotherapy, we have moderate confidence in the results observed for primary prevention and from the payers’ perspective, as detailed in Appendix IV in S1 File.

## Discussion

The current study synthesized the cost-effectiveness of Ezetimibe with statin compared to other lipid-lowering therapeutic agents or placebo using systematic review and meta-analysis of cost-utility studies. Economic evaluation studies are difficult to synthesise due to the differences in economic parameters, income thresholds, study perspectives, costs. Many studies that report cost-effectiveness ignore the CI of the ICER point estimates. To address these issues, we tried to standardise data extraction and preprocessing from various published studies to produce a pooled INB with CI.

The pooled INBs from 25 comparisons identified from 21 studies for the meta-analysis show that EPS therapy is more cost-effective than other lipid-lowering therapeutic agents such as statins, PCSK9i, placebo, or no treatment. Subgroup analysis strengthened the robustness of our findings. We conducted a subgroup analysis to understand the considerable heterogeneity. EPS therapy is significantly cost-effective compared to PCSK9i plus statin and PCSK9i plus Ezetimibe with statins. A plethora of evidence has shown that the main reasons for the underuse of statins in LMICs and UMCs by eligible patients with established CVD [65, 66] were lack of availability, accessibility, and affordability. The subgroup analysis also revealed that EPS therapy is cost-effective against therapeutic agents or placebo in HICs, with payers’ perspective, lifetime horizon, and primary prevention. However, the results lose their robustness and become not significantly cost-effective for HICs and lifetime horizons when we limit the comparator to statin monotherapy alone in further subgroup analysis.

Previous systematic review by Marquina et al. [67], had indicated that Ezetimibe was cost-effective in 62.5% of the included studies, and Suh et al. [68], indicated that Ezetimibe was cost-effective for stain intolerant patients with chronic kidney disease. From the current evidence, we conclude EPS therapy is cost-effective compared to other lipid-lowering therapeutic agents or placebo in HICs, from the payers’ perspective, for primary prevention and the lifetime horizon. However, EPS therapy is not cost-effective for secondary prevention, similar to previously published studies that suggested that PCSK9i may become cost-effective for secondary prevention [63] only if the WTP threshold is increased or if the drug cost is lowered [46, 62]. It is evident that the cost-effectiveness of PCSK9i is increasing with a progressive decrease in drug prices [46, 59, 62].

Bempedoic acid and inclisiran are now available as newer alternatives for lowering cholesterol. Although more expensive, a recent evidence synthesis concluded that using bempedoic acid in combination with or without ezetimibe and inclisiran was cost-effective for HeFH and secondary prevention of ASCVD in patients who required additional LDL-C lowering despite being on maximally tolerated statin therapy [69]. Additional ezetimibe trials are required for bempedoic acid or inclisiran compared with statins plus ezetimibe clinical and economic outcomes to understand the true incremental value of these two new agents, as well as their associated value-based pricing calculations.

For Ezetimibe, the main cost-effectiveness drivers were baseline cardiovascular risks [26, 47, 48, 50, 53, 57], cost of the drug [46, 54, 56, 62], treatment effects related to cardiovascular and non-cardiovascular mortality [28, 54], and time horizon [46, 57, 61]. For some other studies, the cost-effectiveness of EPS therapy compared with statin monotherapy was subject to certain conditions. Davies et al. [54] reported that EPS therapy was cost-effective for secondary prevention of CHD and stroke. Also, the study reported that for primary prevention of CHD and stroke in patients whose LDL-C levels were > 100 mg/dL and in patients with diabetes, Ezetimibe becomes cost-effective only if we consider a 90% cost reduction. Soini et al. [51] showed that EPS therapy is cost-effective only in specific sub-populations of men and diabetic women. A study by Ara et al. [48] reported that EPS therapy becomes cost-effective in the UK health system when using a threshold of £30,000 per QALY instead of the £20,000 per QALY value. Similarly, Almalki et al. [57] reported that EPS therapy was not cost-effective for 5 and 10 year models but became a cost-effective treatment when a lifetime horizon is used.

The drug price and WTP per additional QALY play an important role in determining the cost-effectiveness of EPS therapy. The differences observed in reported outcomes and the high heterogeneity estimated among the studies may be the result of changes in drug prices or cost estimations under different study perspectives. This necessitates the need for context-specific future research in primary economic evaluations for EPS therapy compared with different lipid-lowering therapeutic agents with a more accurate estimate of costs. More studies from LMICs and societal perspectives are also needed for the generalisability of results.

The limitation of our study is that the comprehensiveness of this cost-effectiveness results is arguable since most clinical data comes from western countries, which are set in HICs, which determine the cost of long-term treatment and prescriptions. Only a small percentage comes from Asian studies and none from Africa, Australia, or South America, indicating the need for more studies using LMICs and societal perspectives. A majority of the studies did not include indirect costs, and none captured the real-world burden of CVD or considered out-of-pocket expenditure or caregiver time and costs. The use of surrogate markers even when clinical endpoints are recommended [70], shows lack of clinical trials describing complex outcomes and have increased the uncertainties in the models. The use of clinical trials to model event rates could lead to an underestimation of the baseline risk and the treatment effect, as shown in Lindh et al. [71]. The mean age reported in majority of the studies was above 60 years. Although LDL-C poses a cumulative risk, lowering LDL-C levels does not always result in a reduction in cardiovascular risk, and prolonged exposure to lower LDL-C from an early point in life substantially reduces the risk of CHD [72]. Given the focus on populations with established CVD, most studies did not examine the effects of treatment initiation at different ages. Moreover, some models used statin trial data to model baseline cardiovascular risk, while others used local demographic data.

Another limitation is that we used funnel plots to examine publication bias because we had no specific measures for non-normally distributed INB. We used the GRADE approach to assess the outcome quality because there were no specific GRADE guidelines for cost-utility studies, and the current approach had its limitations [73]. We could not discern a clear trend regarding whether using data from clinical trials instead of observational studies in terms of baseline risk and treatment effect improves the cost-effectiveness results. The structural variation among studies could also raise the overall conclusion’s uncertainty. Many of these models are not publicly available which limits the ability to compare between settings and countries [74]. Only four studies [46, 55, 61, 62] included adverse events. Adherence was not modelled in any studies, even when lower adherence rates have been shown to result in poorer health outcomes and higher costs for healthcare systems time after time [75]. Further, due to limited information provided, the costs for co-morbidities and gender differences could not be explored. When extrapolating to other healthcare contexts, the generalisability of these results should be done with careful consideration. For LICs and LMICs, we suggest generation of primary economic evidence to guide the policy decision, however till then the evidence based on the clinical effectiveness may guide the stake holders in their decision.

## Conclusion

This systematic review and meta-analysis concluded that EPS therapy is a significant cost-effective option compared to other lipid-lowering therapeutic agents or placebo. The subgroup analysis supported the findings in HICs, from payers’ perspective, for primary prevention and for the lifetime horizon. However, the robustness of the results is lost for HICs and lifetime horizon when EPS therapy is compared with statin monotherapy alone, where it is not significantly cost-effective. The GRADE quality assessment revealed a low confidence in the results observed for EPS therapy compared to other lipid-lowering therapeutic agents or placebo and statin monotherapy. The majority of the studies were from HICs and undertook a healthcare perspective, highlighting a lacuna in evidence to be filled from a societal perspective and LMICs.

## Supporting information

S1 Checklist PRISMA checklist. (DOCX)

S1 File Online supplementary material. (PDF)
