# Supplementary material for: Cost-effectiveness of Ezetimibe plus statin lipid-lowering therapy: A systematic review and meta-analysis of cost-utility studies
Source: PLoS One. 2022 Jun 16;17(6):e0264563. doi: 10.1371/journal.pone.0264563 (PMC9202874; doi:10.1371/journal.pone.0264563)
Supplement: S1 File — (PDF) [file pone.0264563.s002.pdf]

Supplementary Material

Of manuscript:

Sasidharan A., Sajith Kumar S., Jagadeesh K.V., Natarajan M., Bagepally B.S. “Cost-effectiveness of Ezetimibe plus statin lipid-lowering therapy: A systematic review and meta-analysis of cost-utility studies”

- **Supplementary Figures**
- **Supplementary Tables**
- **Appendix I:** Search Strategy
- **Appendix II:** Methods
- **Appendix III:** Summary of Findings of GRADE Assessment

Supplementary Figure 1- Assessment of Risk of Bias using ECOBIAS Checklist

Author Year / Issue addressed	Kohli_2006	Ara_2008(1)	Ara_2008(2)	Ara_2008(3)	John Reckless_2010	Erkki Soini_2010	Noten_2011	Laires_2015	Mihaylova_2016	Kazi_2016	Kazi_2017	Almalki_2017	Davies_2017	Stam-Slob _2017	Korman_2018	Stam-Slob _2018	Kongpakwattana_2019	Kazi_2019	Schlackow_2019	Dressel_2019	Landmesser_2020	Han Yang_2020
Narrow perspective bias	Y	N	Y	Y	Y	Y	Y	Y	N	Y	Y	Y	Y	Y	Y	Y	Y	Y	Y	Y	Y	Y
Inefficient comparator bias	Y	Y	Y	Y	Y	Y	Y	Y	Y	Y	Y	Y	Y	Y	P	Y	Y	Y	Y	Y	Y	Y
Cost measurement omission bias	P	Y	Y	Y	P	Y	P	Y	Y	P	P	Y	Y	P	Y	P	Y	P	Y	Y	Y	P
Intermittent data collection bias	U	U	U	U	Y	Y	U	U	Y	U	U	U	Y	U	Y	U	Y	U	U	Y	Y	U
Invalid valuation bias	Y	Y	Y	Y	Y	Y	P	Y	Y	U	U	Y	Y	P	P	P	Y	U	Y	P	Y	Y
Ordinal ICER bias	Y	Y	Y	Y	Y	Y	Y	Y	Y	Y	Y	Y	Y	Y	Y	Y	Y	Y	Y	Y	Y	Y
Double-counting bias	U	U	U	U	Y	Y	U	U	U	U	U	U	U	U	U	U	U	U	U	P	Y	U
Inappropriate discounting bias	Y	Y	Y	Y	Y	Y	Y	Y	Y	Y	Y	Y	Y	Y	Y	Y	Y	Y	Y	Y	Y	Y
Limited sensitivity analysis bias	P	P	Y	Y	P	Y	P	P	P	P	P	P	P	P	P	P	Y	P	P	P	P	P
Sponsor bias	N	Y	Y	Y	Y	Y	Y	Y	Y	Y	Y	Y	Y	Y	Y	Y	Y	Y	Y	N	Y	N
Reporting and dissemination bias	NA	NA	U	U	Y	U	Y	NA	Y	U	U	Y	Y	Y	U	Y	U	U	Y	Y	P	NA
Structural assumptions bias	Y	Y	Y	Y	Y	Y	U	Y	U	Y	Y	Y	P	U	Y	U	Y	Y	Y	Y	Y	Y
No treatment comparator bias	Y	Y	Y	Y	Y	Y	Y	Y	Y	Y	Y	Y	Y	Y	Y	Y	Y	Y	Y	Y	Y	Y
Wrong model bias	Y	Y	Y	Y	Y	Y	Y	Y	U	Y	Y	Y	Y	Y	Y	Y	Y	Y	Y	Y	Y	Y
Limited time horizon bias	Y	Y	Y	Y	Y	Y	Y	Y	N	Y	Y	Y	Y	Y	Y	Y	Y	Y	Y	Y	Y	Y
Bias related to data identification	Y	Y	Y	Y	Y	Y	Y	Y	Y	Y	Y	Y	Y	Y	P	Y	Y	Y	Y	Y	Y	Y
Bias related to baseline data	Y	Y	U	U	Y	Y	P	U	NA	Y	Y	Y	Y	P	P	P	Y	Y	U	Y	Y	Y
Bias related to treatment effects	P	Y	Y	Y	Y	Y	P	U	P	P	P	P	P	P	P	P	Y	P	U	Y	Y	P
Bias related to quality-of-life weights	Y	Y	Y	Y	Y	Y	Y	Y	Y	Y	Y	Y	Y	Y	Y	Y	Y	Y	Y	Y	Y	Y
Non-transparent data incorporation bias	Y	Y	Y	Y	Y	Y	Y	Y	Y	Y	Y	Y	Y	Y	Y	Y	Y	Y	Y	Y	Y	Y
Limited scope bias	P	P	Y	Y	Y	Y	P	P	P	P	P	P	P	P	P	P	P	P	P	P	P	P
Bias related to internal consistency	U	U	U	U	Y	Y	U	U	U	U	U	U	U	U	U	U	U	U	U	U	U	U

Y- Yes, N-No, P-Partly, U-Unclear, NA- Not Applicable | Source: http://dx.doi.org/10.1586/14737167.2015.1103185

Supplementary Figure 2- Funnel plot and contour-enhanced funnel plot to distinguish publication bias

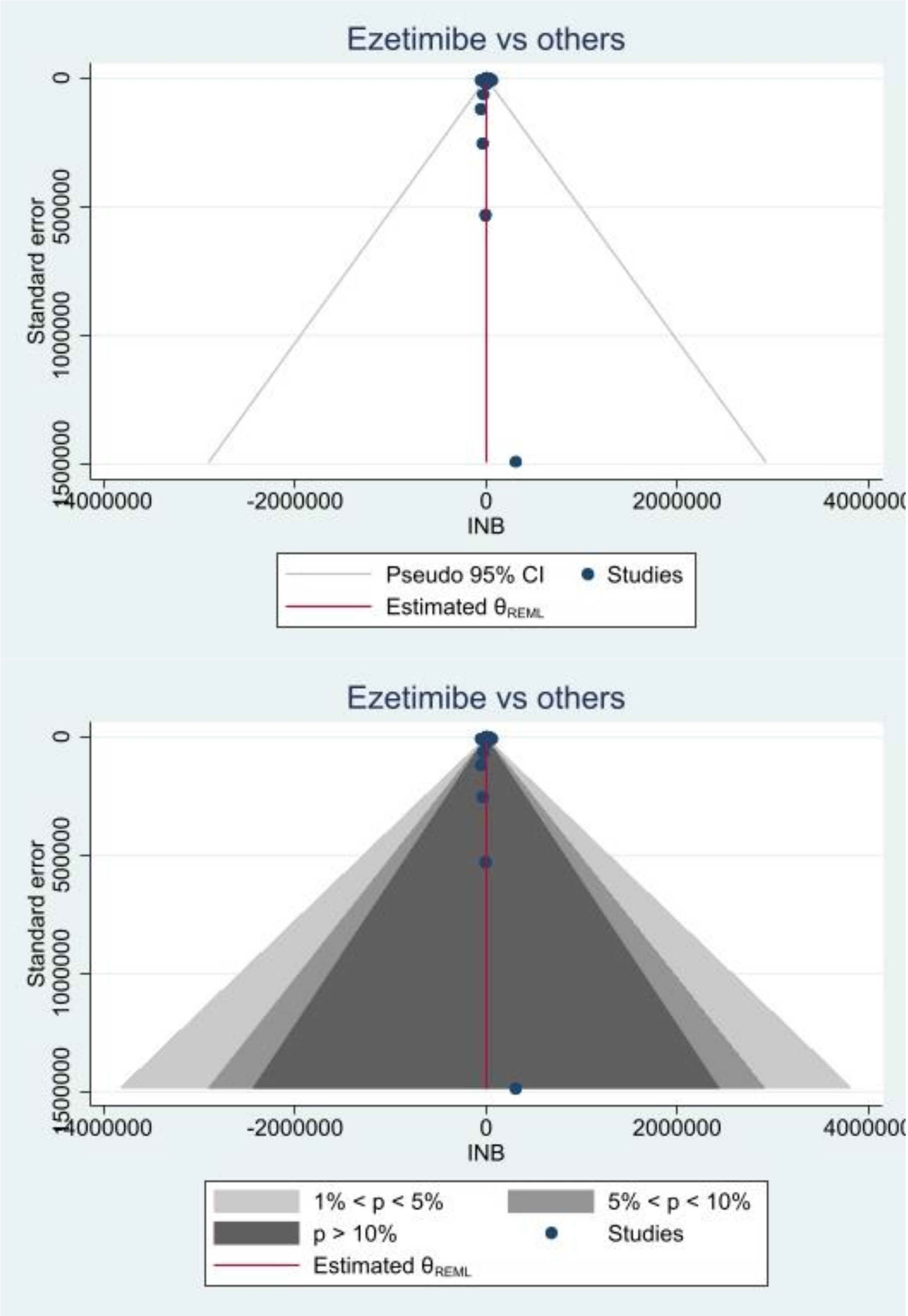

Supplementary Figure 3- Subgroup analysis of pooled INBs based on different Treatment Group

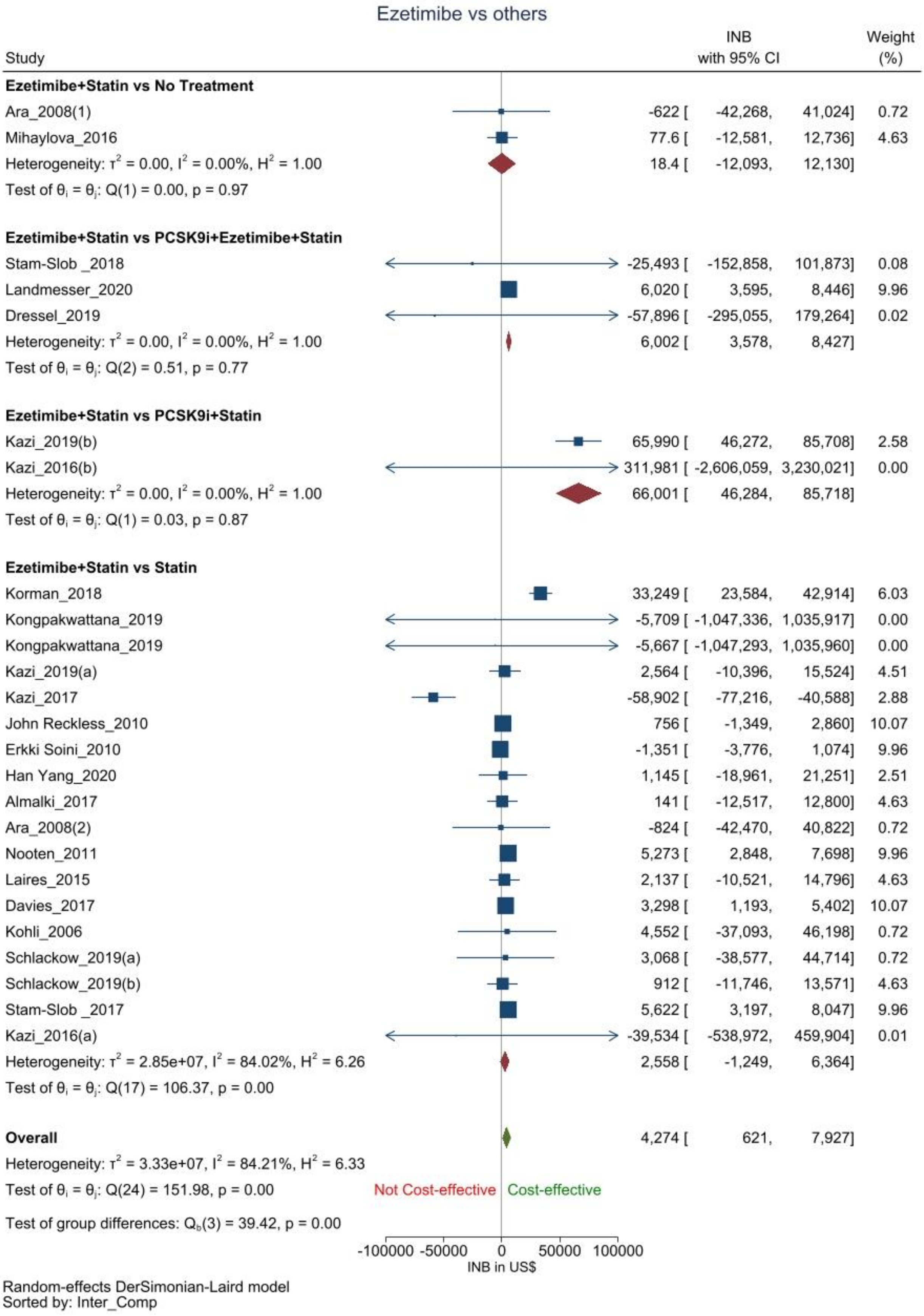

Supplementary Figure 4- Subgroup analysis of pooled INBs based on Income classification

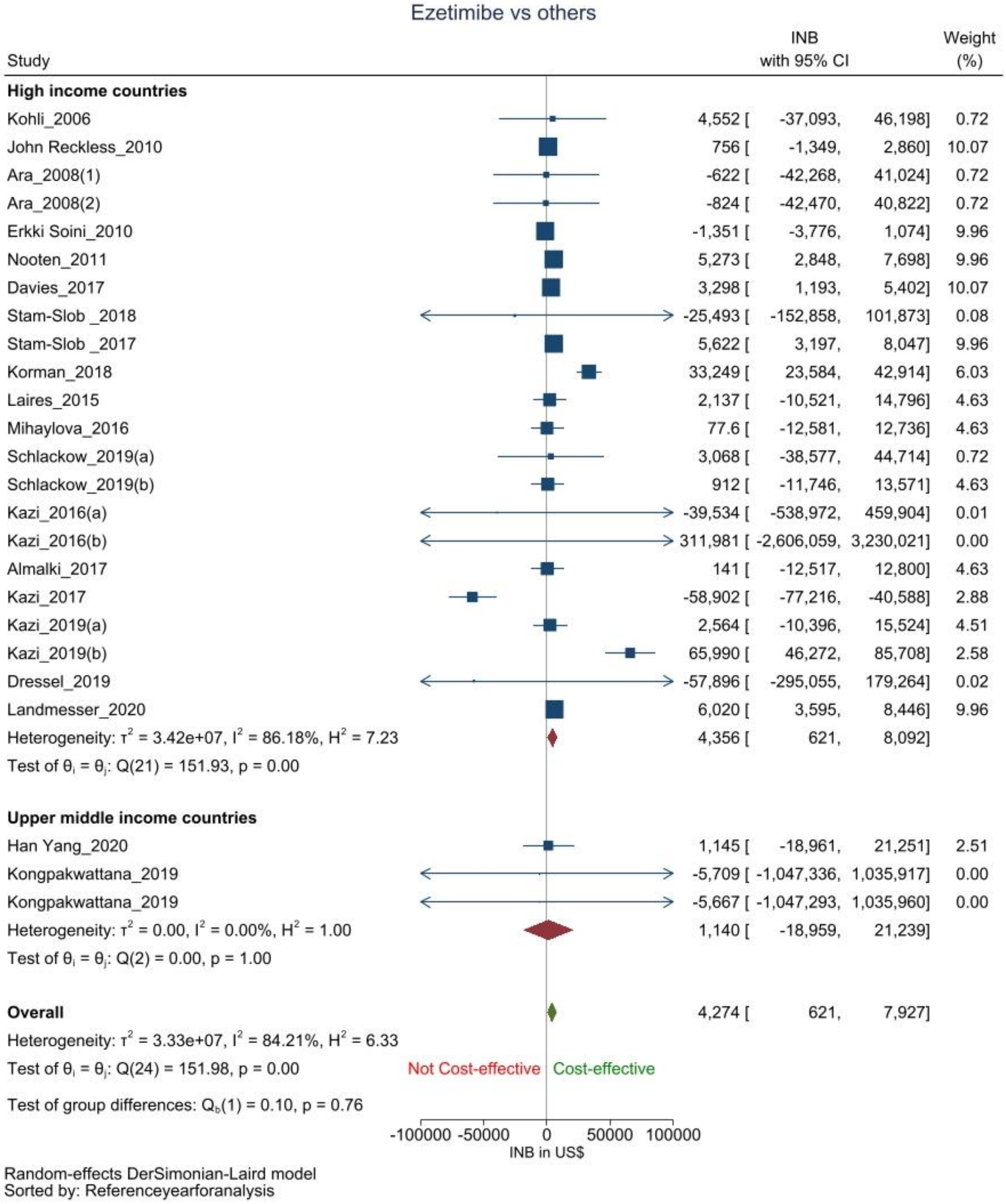

Supplementary Figure 5- Subgroup analysis of pooled INBs based on study perspective

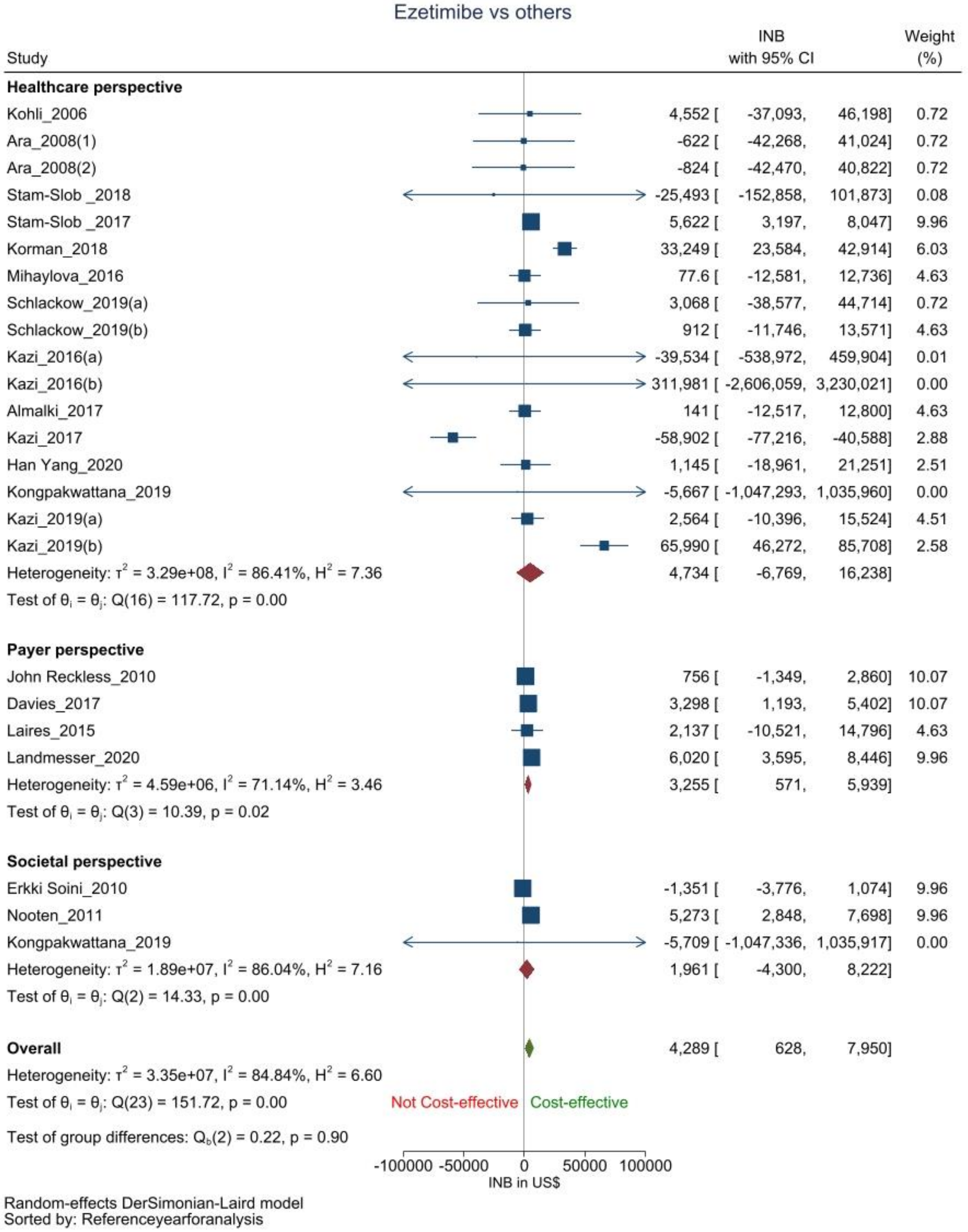

Supplementary Figure 6- Subgroup analysis of pooled INBs based on Time horizon

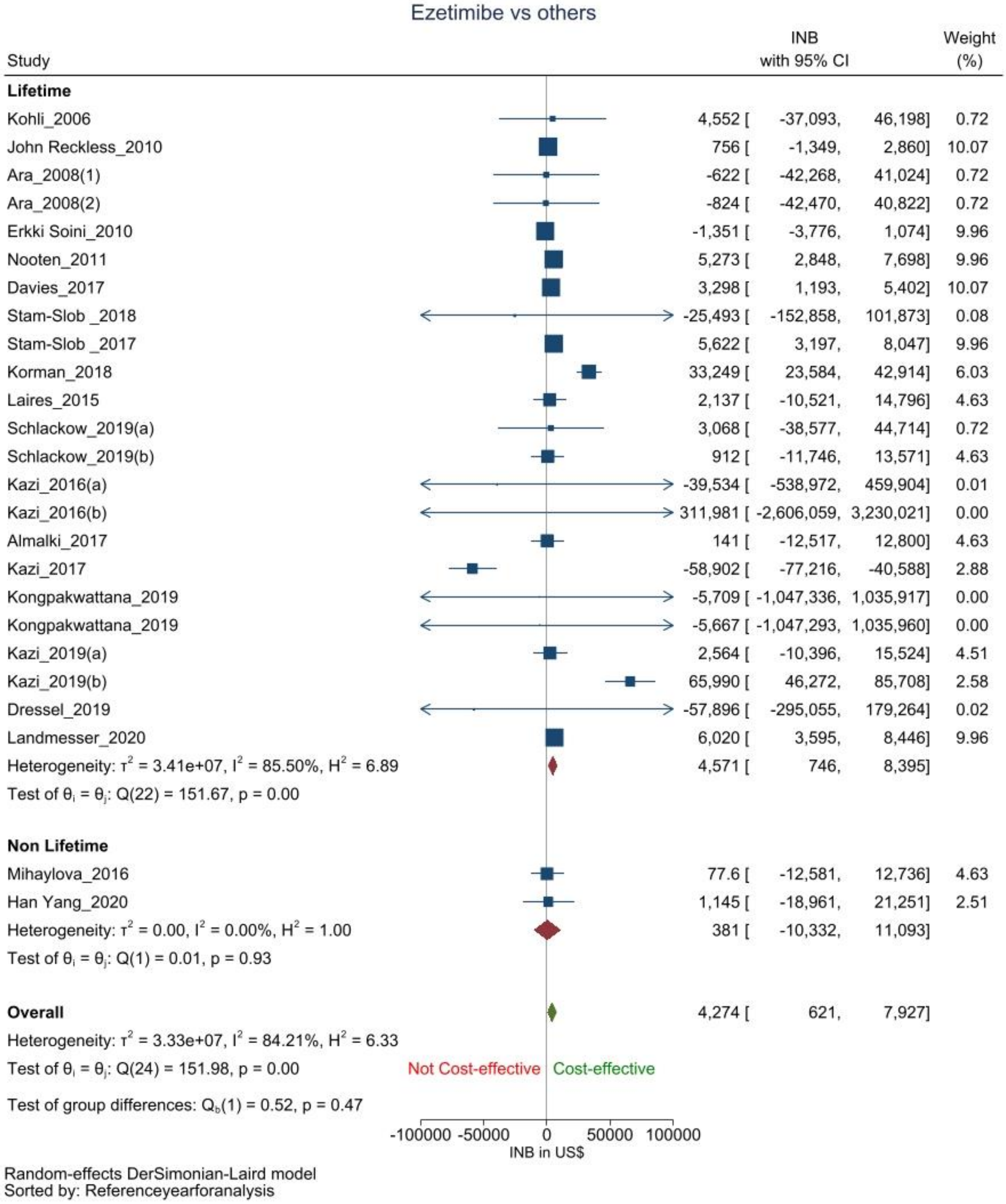

Supplementary Figure 7- Subgroup analysis of pooled INBs based on discount rate

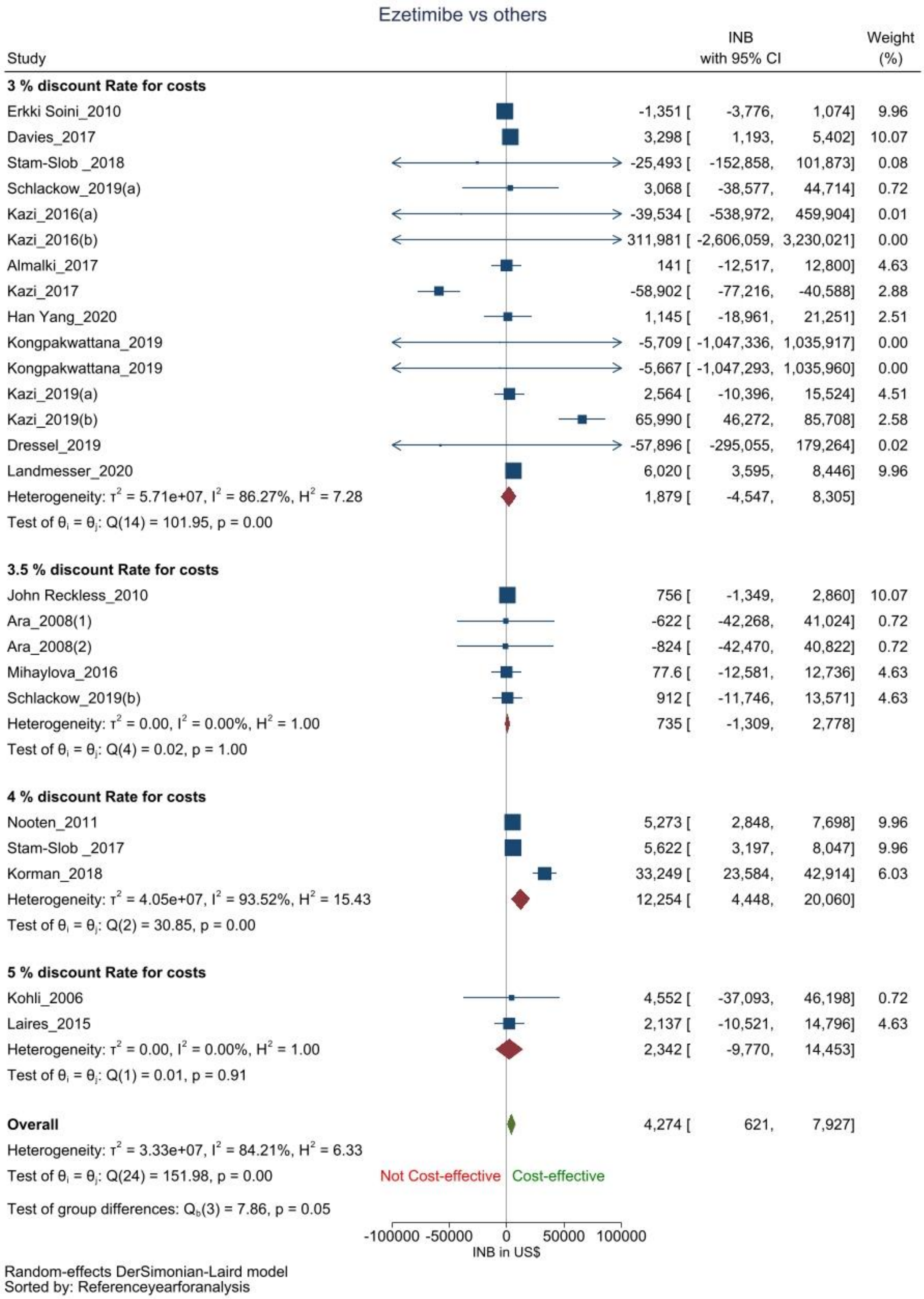

Supplementary Figure 8- Subgroup analysis of pooled INBs based on disease prevention

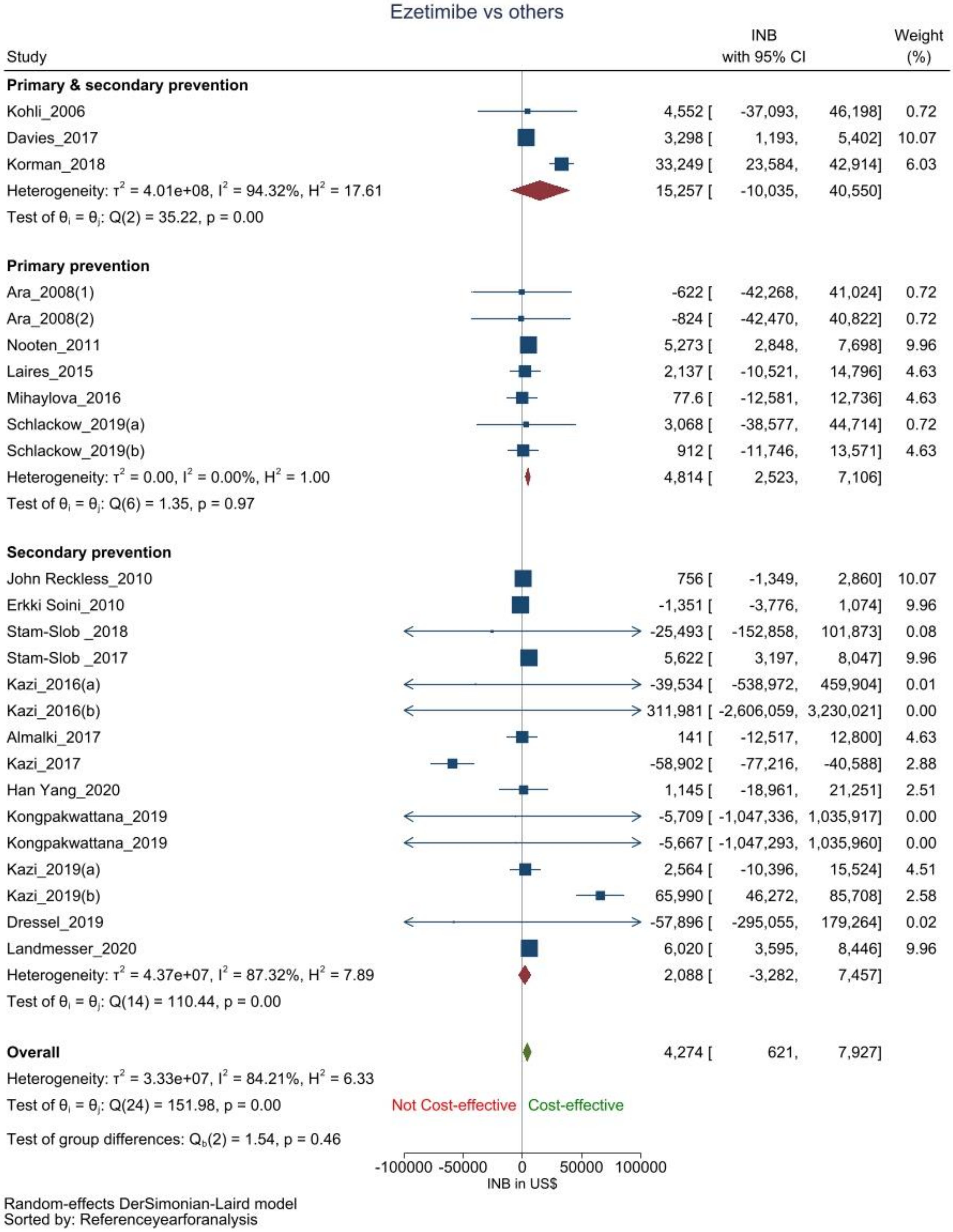

(1)

Supplementary Figure 9- Subgroup analysis of pooled INBs based on threshold

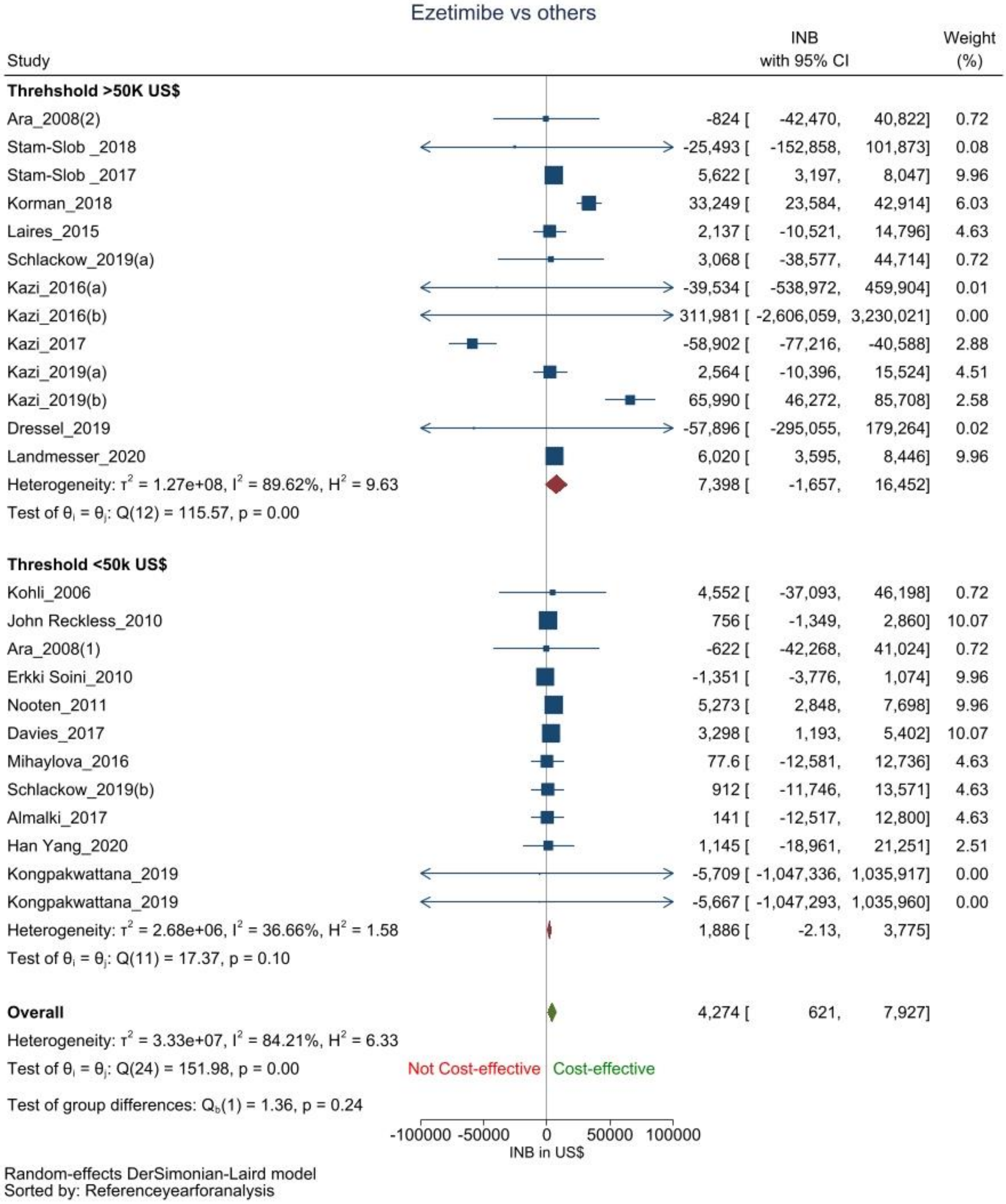

Supplementary Figure 10- Subgroup analysis of pooled INBs based on different scenario

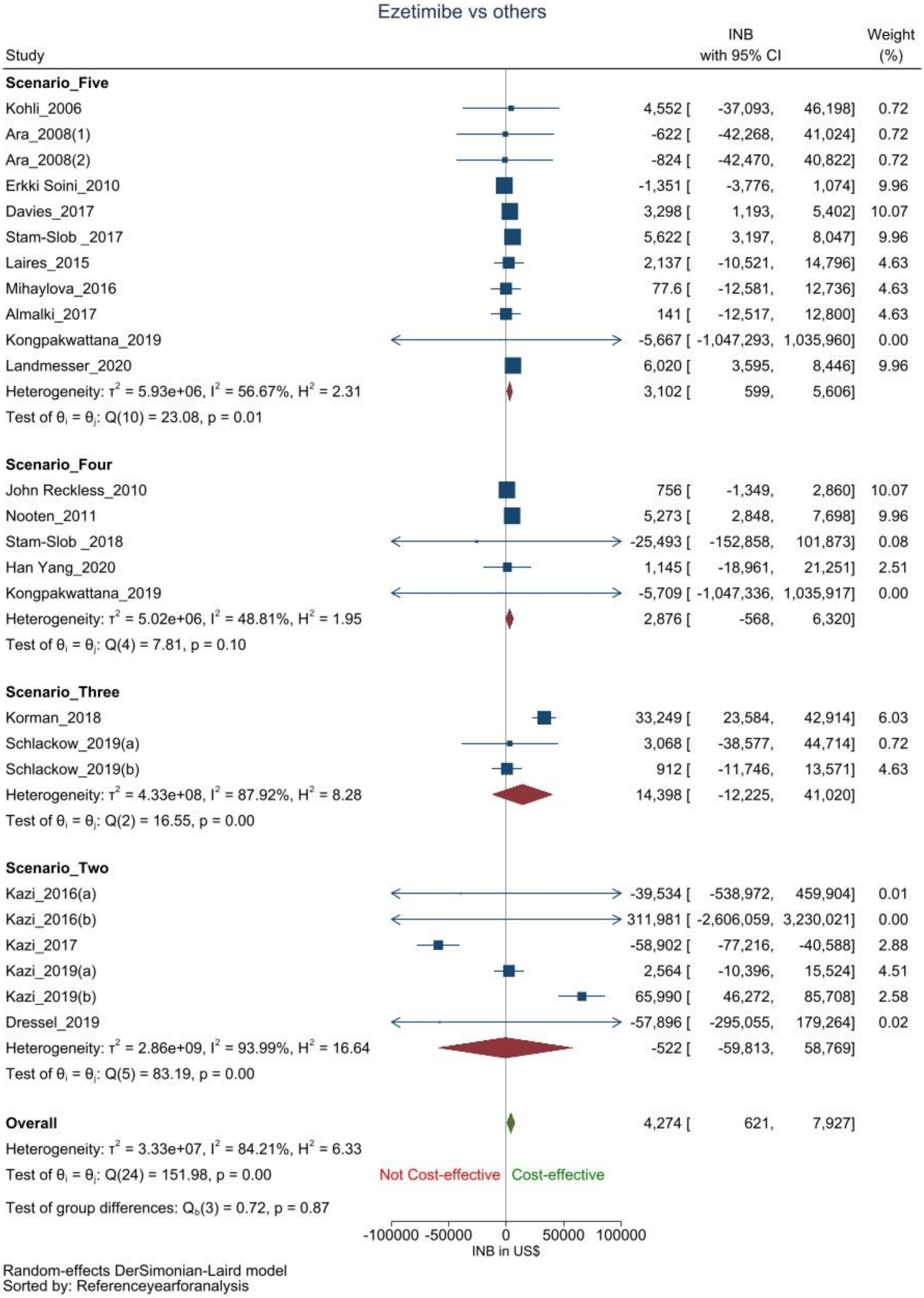

Supplementary Figure 11- Sensitivity analysis of Pooled INBs based on Prevention

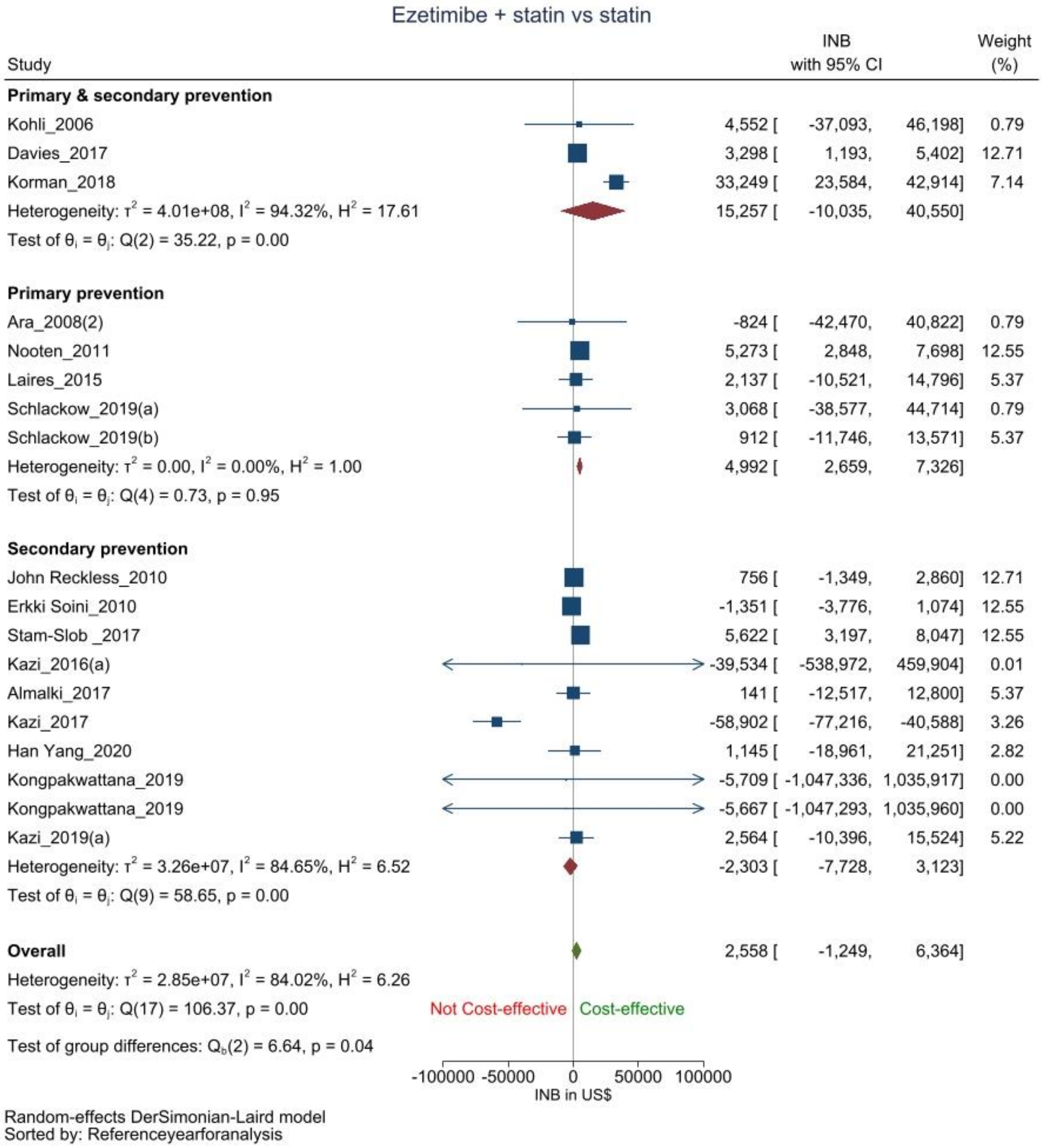

Supplementary Figure 12- Sensitivity analysis of Pooled INBs based on study perspective

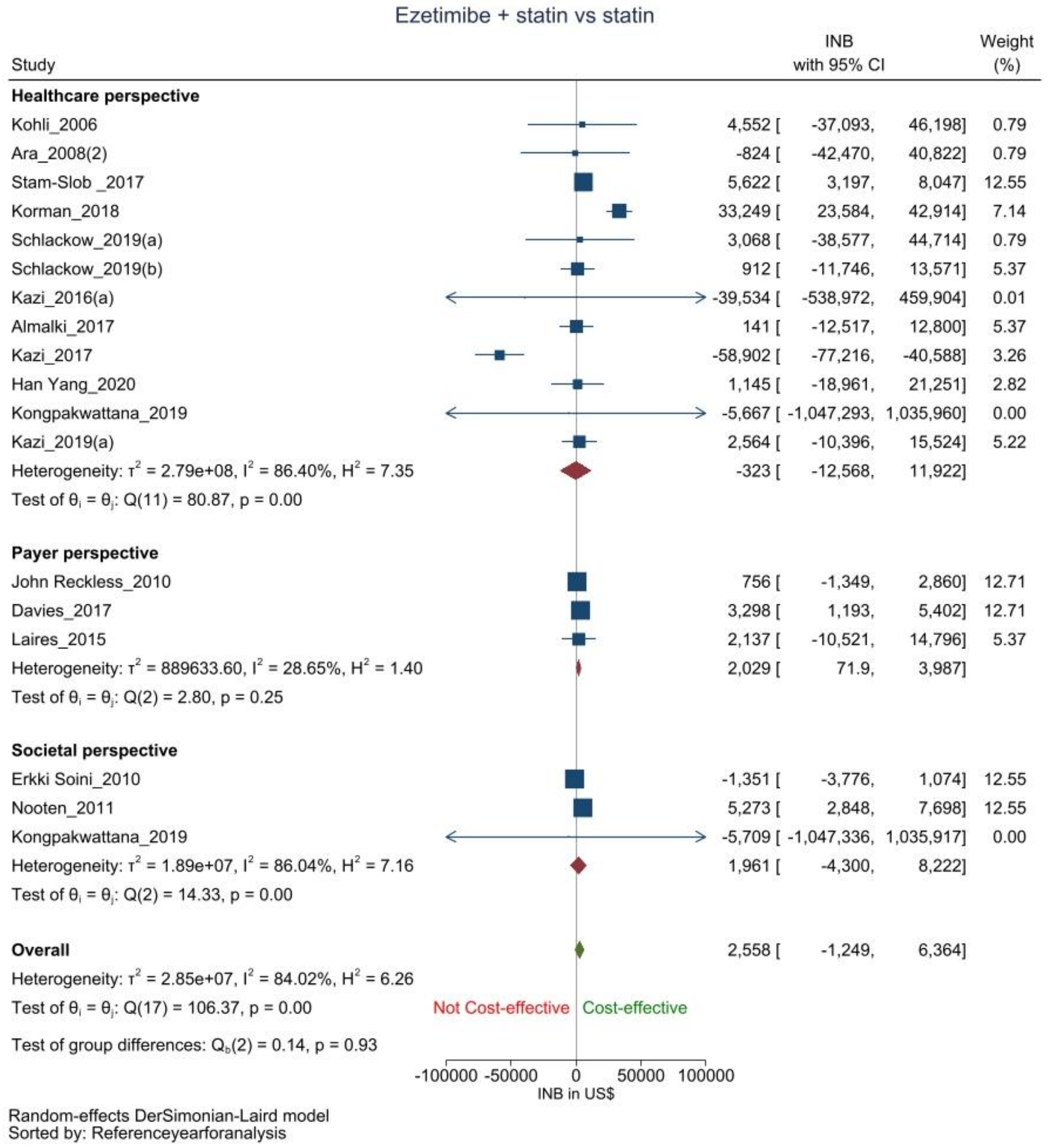

Supplementary Figure 13- Sensitivity analysis of Pooled INBs based on Income classification

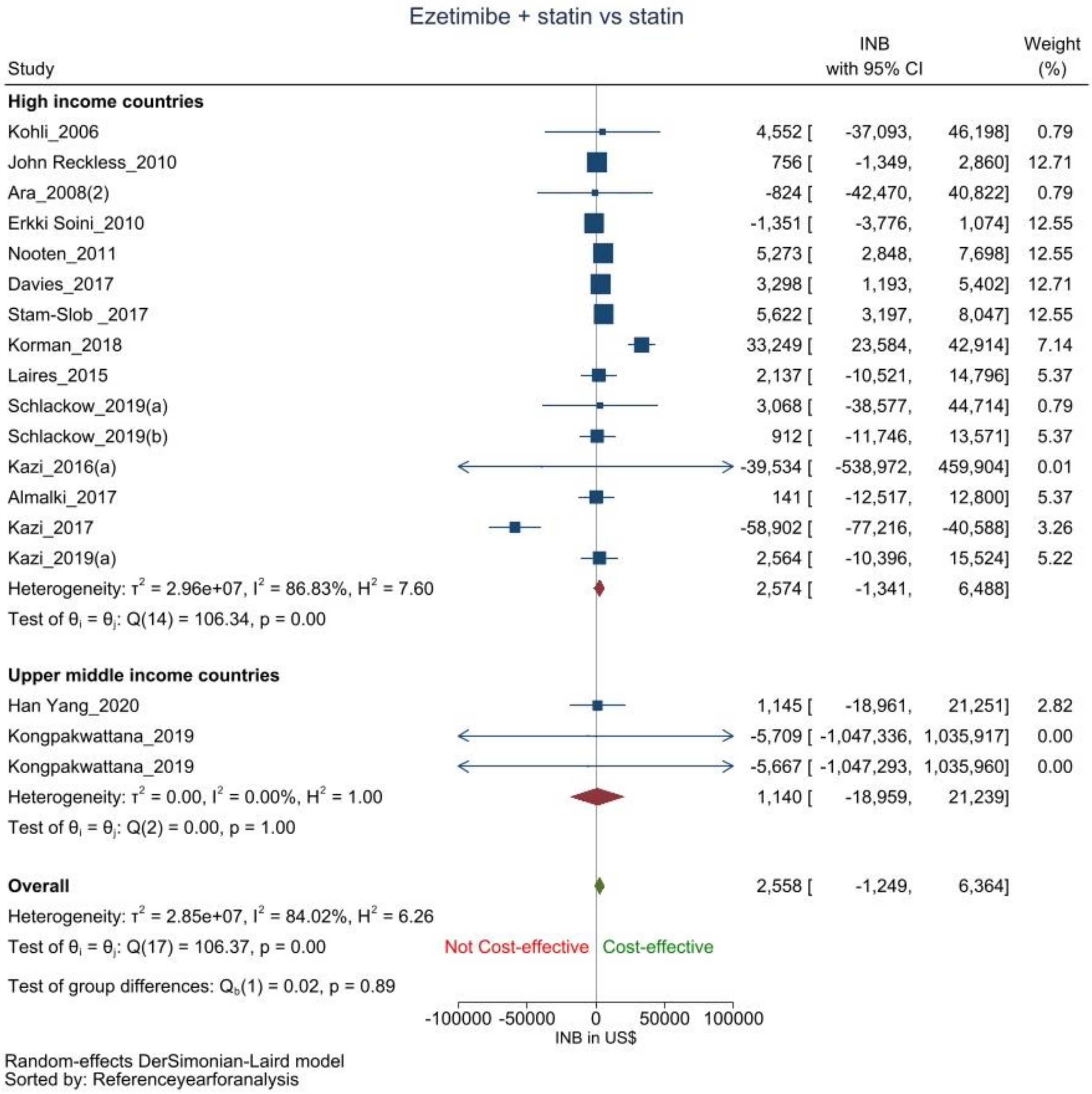

Supplementary Figure 14- Sensitivity analysis of Pooled INBs based on time horizon

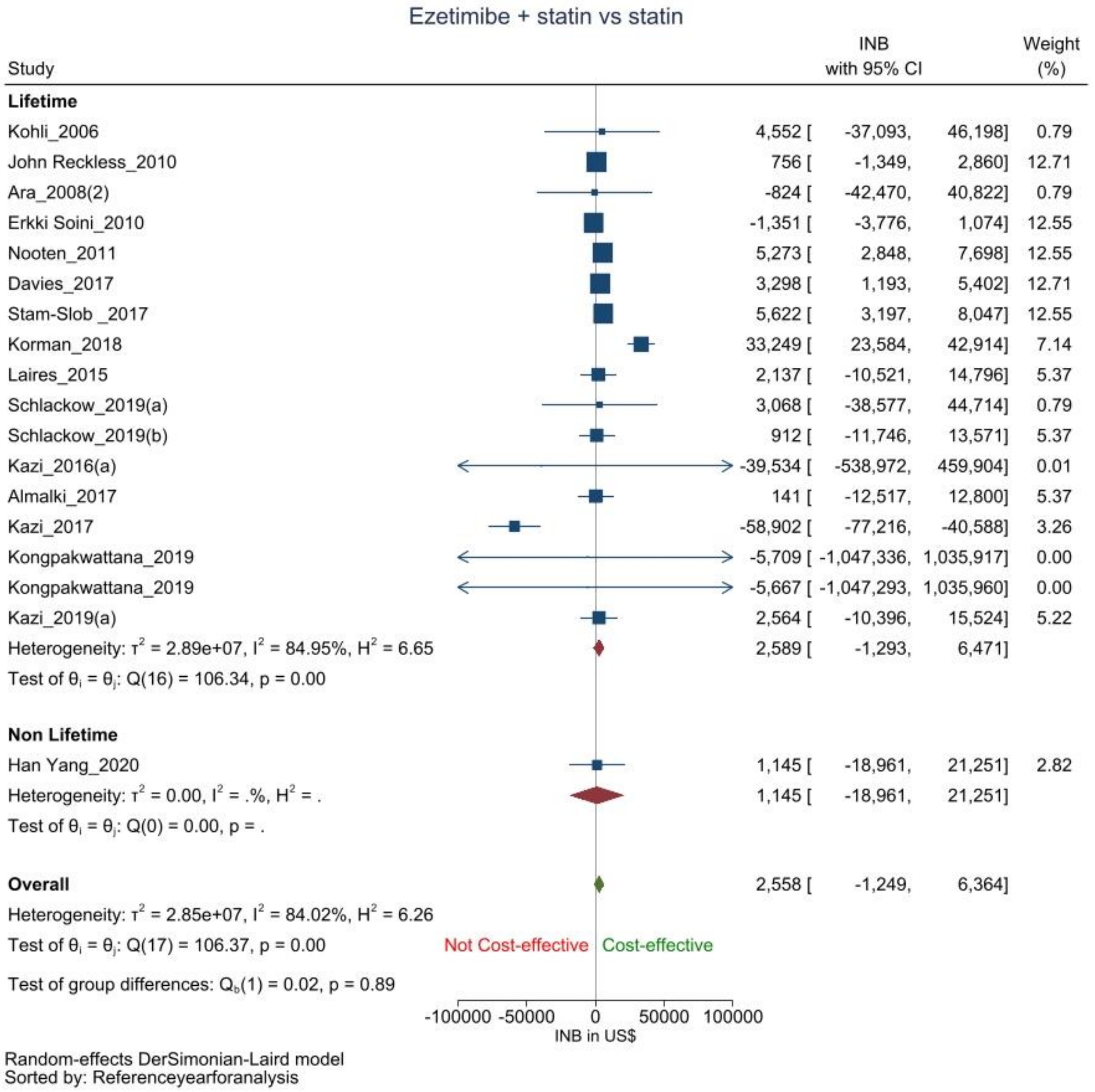

Supplementary Table 1- Results of subgroup analysis of INBp of Ezetimibe vs Others and Ezetimibe vs Statin monotherapy

	**Sub-group (no. of comparisons)**	**INB (CI) US$**	**I^2 (%)**
**Ezetimibe vs Others**	Overall (25)		84.21
Country	Canada (1)	4,552 (-37,093 to 46,198)	
	China (1)	1,145 (-18,961 to 21,251)	
	Finland (1)	-1,351 (-3,776 to 1,074)	
	Germany (1)	-57,896 (-295,055 to 179,264)	
	Netherlands (3)	5,442 (3727 to 7,157)	
	Norway (1)	33,249 (23,584 to 42,914)	
	Portugal (1)	2,137 (-10,521 to 14,796)	
	Saudi Arabia (1)	141 (-12,517 to 12,800)	
	Sweden (1)	6,020 (3,596 to 8,446)	
	Thailand (2)	-5688 (-742,229 to 730,853)	
	United Kingdom (4)	392 (-8,172 to 8,956)	
	United States of America (8)	1,923 (-7,384 to 11,299)	
**Ezetimibe vs Statin monotherapy**	Overall (18)	2,557 (-1,249 to 6,364)	84.02
**Income classification**	HIC (15)	2,574 (-1,341 to 6,488)	86.83
	UMIC (3)	1,140 (-18,959 to 21,239)	0.00
**Study perspective**	Healthcare perspective (12)	-323 (-12,568 to 11,922)	86.40
	Payer perspective (3)	2,029 (71.9 to 3,987)	28.65
	Societal perspective (3)	1,961 (-4,300 to 8,222)	86.04
**Prevention**	Primary & Secondary Prevention (3)	15,257 (-10,035 to 40,550)	94.32
	Primary Prevention (5)	4,992 (2,659 to 7,326)	0.00
	Secondary Prevention (10)	-2,303 (-7,728 to 3,123)	84.65
**Time horizon**	Lifetime horizon (17)	2,589 (-1,293 to 6,471)	84.95
	Non-lifetime horizon (1)	1,145 (-18,961 to 21,251)	0.00

APPENDIX I

SEARCH STRATEGIES

	Pubmed Search	Hits as on 26-04-2021
I	Ezetimibe OR Zetia OR Ezetrol OR ezetimib OR ezetrol OR ‘sch 58235’ OR sch58235 OR zient	3,768
O	“quality of life” OR “QALY” OR “quality adjusted” OR “life year” OR “life years” OR “DALY” OR “disability adjusted” OR “cost effective” OR utility OR “ICER” OR “ICERS” OR INB OR "economics"[MeSH Terms] OR "economics, pharmaceutical"[MeSH Terms]	4,493,535
	P & O	650
	Embase Search	
I	'ezetimibe'/exp OR '1 (4 fluorophenyl) 3 [3 (4 fluorophenyl) 3 hydroxypropyl] 4 (4 hydroxyphenyl) 2 azetidinone' OR 'absorcol' OR 'ezetib' OR 'ezetimib' OR 'ezetimibe' OR 'ezetrol' OR 'sch 58235' OR 'sch58235' OR 'viemm' OR 'zetia' OR 'zient'	11,956
O	'cost benefit analysis'/exp OR 'cost analysis' OR 'cost benefit' OR 'cost benefit analysis' OR 'cost benefit ratio' OR 'cost-benefit analysis' OR 'cost minimization analysis'/exp OR 'cost minimization' OR 'cost minimization analysis' OR ‘quality of life’ OR ‘QALY’ OR ‘quality adjusted’ OR ‘life year’ OR ‘life years’ OR ‘DALY’ OR ‘disability adjusted’ OR ‘ICER’ OR ‘ICERS’ OR INB OR 'cost effectiveness analysis'/exp OR 'cost effectiveness' OR 'cost effectiveness analysis' OR 'cost effectiveness ratio' OR 'cost efficiency analysis' OR ‘willingness to pay’ OR 'cost utility analysis'/exp OR 'cost utility' OR 'cost utility analysis'	894,347
	P & O	612

Appendix II

	Scopus	Hits as on 26-04-2021
	(ezetimibe OR zetia OR ezetrol OR ezetimibe OR ezetimib OR ezetimibe OR ezetrol OR "sch 58235" OR sch58235 OR zetia OR zient) AND ("cost effectiv*" OR "cost utility" OR "cost benefit" OR "cost-benefit" OR "quality adjusted life years" OR qaly OR ly OR "life year$" OR daly OR "disability adjusted" OR "incremental cost effective ratio" OR "ICER" OR "incremental net benefit" OR inb OR "benefit ratio" OR 'cost AND benefit' OR 'cost AND minimi?ation' OR "cost-effectiveness" OR "cost effectiveness ratio" OR "cost efficiency analys?s" OR "cost utility") AND (LIMIT-TO (DOCTYPE , "ar"))	682

APPENDIX II: Methods

A) Estimation of incremental net benefit Incremental net benefit (INB) can be estimated as follows:

$$INB = Kx\Delta E - \Delta C ----------------- (1)$$

or

$$INB = \Delta E(K - ICER) ------------------(2)$$

$$Var(INB) = K^2\, \sigma_{\Delta E}^2 + \sigma_{ICER}^2 ------------ (3)$$

or

$$Var(INB) = K^2\sigma_{\Delta E}^2 + \sigma_{\Delta C}^2 - 2K\rho_{\Delta E \Delta C} ------- (4)$$

K is the Willingness to pay (WTP), ΔC and ΔE are incremental cost and incremental effectiveness, $\sigma^2_{\Delta C}$, $\sigma^2_{\Delta E,}$ $\rho_{\Delta C \Delta E}$ were variances of ΔC and ΔE and their covariance, and σ^2_{ICER} was variance of ICER. The WTP was used as reported in the original included studies, i.e., a standard/country specific or GDP based WTP threshold. A positive INB favours treatment, i.e., intervention is cost-effective, whereas a negative INB favours the comparator, i.e., intervention is not cost-effective.

Currency conversions and standardization

The monetary units were converted to purchasing power parity (PPP), adjusted to US$ for the year 2021 before INB calculation. For instance, if a study reported cost, ICER, and thresholds in Euros for 2012, this currency was first converted to 2021 Euros using the historical consumer price index (CPI) of that country. The Euro 2021 value was next converted to PPP adjusted US$ rate using conversion rates from the International Monetary Fund[25]. In addition, the K value from GDP-based threshold was corrected for both latest CPI (2021) and PPP, while for standard/country specific or fixed K, only PPP was corrected. For the variance monetary value conversion, the specific study variance was multiplied by the square of total factors (i.e., CPI and PPP) for the year

2021. For example, if Y is variance of ICER in Euros 2012, this was converted into 2021 PPP adjusted US$ as

$$Var_{PPP_{2021}} = Var_{Euros_{2012}} x \left(\frac{CPI_{Euros_{2021}}}{CPI_{Euros_{2012}}} x \frac{1}{PPP_{2021}} \right)^2 ------(5)$$

B) Meta-analysis

i. A fixed effect model

$$\mathrm{INB_p} = \frac{\sum_{i=1}^{S} w_i.\mathrm{INB_i}}{\sum_{i=1}^{S} w_i} ----------------------(1)$$

$$w_i = \frac{1}{Var(INB_i)} ----------------------------(2)$$

ii. A random effect model

$$\mathrm{INB_p} = \frac{\sum_{i=1}^{S} w_i^*.\mathrm{INB_i}}{\sum_{i=1}^{S} w_i^*} ----------------------(3)$$

$$w_i^* = \frac{1}{Var(INB_i)+\tau^2} -----------------------(4)$$

$$\tau^2 = \frac{Q-(S-1)}{\sum w_i - \frac{\sum w_i^2}{\sum w_i}} ---------------------------(5)$$

Q is the Cochrane Q-statistic, where Q = 0 if Q < S-1; and s is the number of included studies/comparisons. The heterogeneity of INB was assessed using Cochrane Q-test and I^2 statistic calculated as equations below.

$$\mathrm{Q} = \sum_{i=1}^{S} w_i(\mathrm{INB_i} - \mathrm{INB}_p)^2 -------------(6)$$

$$\mathrm{I}^2 = 100\%\mathrm{x}\ \frac{\mathrm{Q-(S-1)}}{\mathrm{Q}} ----------------------(7)$$

C) Scenarios developed to obtain variance

- Scenario-1: studies which reports the point estimates & variances for every parameter required for calculation
- Scenario-2: studies which reports the means and 95% CIs of incremental costs & outcomes, and ICER

$$95\%\ CI\ of\ \mu_{ICER} = \hat{\mu}_{ICER} \pm Z_{\alpha/2} \times SE$$

$$UL_{ICER} = \hat{\mu}_{ICER} \pm Z_{\alpha/2} \times SE$$

$$SE = \frac{UL_{ICER} - \hat{\mu}_{ICER}}{Z_{\alpha/2}}$$

$$\hat{\sigma}^2{}_{ICER} = SE^2$$

UL_{ICER} = Upper limit of ICER

$Z_{\alpha/2}$ = Standard Normal = 1.96

$\hat{\mu}_{ICER}$ = mean ICER

- Scenario-3: studies which reports means and 95% CI of costs/outcomes, or ΔC& ΔE, but not ICER or its variance.

  Monte Carlo simulation with a gamma and normal distributions for ΔC and ΔE is performed to estimate covariance between ΔC and ΔE.

- Scenario-4: studies which does not report any dispersion, but provides the CE plane graphs,

  Data can be directly extracted from the CE plane using Web-Plot Digitizer software. The means of ΔC, ΔE, and their variances and co-variance can be estimated accordingly.

- Scenario-5: The study reports only the means (or point estimates) of costs, outcomes, and ICER.

  The measures of dispersions can be borrowed from another similar study if they fulfil the following criteria:

  - They are in the same stratum of country income level, perspective, intervention, comparator, time period, country region, model type, and inputs (i.e., discounting, time horizon).
  - Their ICERs are not much different, e.g., ±50% to 75%

Appendix III: Summary of Findings of GRADE Assessment

Evidence Profile using Grading of Recommendation, Assessment, Development, and Evaluation (GRADE) instrument

P: Adult subjects requiring the lipid-lowering therapy
I: Ezetimibe with statin therapy
C: Any other lipid-lowering therapeutic agents/placebo
O: economic outcomes of incremental cost-effectiveness ratio (ICER), quality-adjusted life years (QALY), or INB.

**Outcome: Cost-effectiveness (assessed with meta-analysis of cost utility analysis)**									
**Quality assessment***						**Summary of findings**			**Comments**
**No of studies**	**Risk of Bias**	**Inconsistency**	**Indirectness**	**Imprecision**	**Publication Bias**	**Effect (*10^3 US$)**		**Certainty/Quality**	
						**INB**	**95%CI**		
Cost-effectiveness of ezetimibe with statin therapy when compared to other lipid-lowering therapeutic agents/placebo (Assessed with meta-analysis).									
21	not serious	serious[a]	serious[d]	not serious	Publication bias is unlikely but identifying publication bias or is due to other causes would be challenging due to high between-study heterogeneity.	4.27	(0.62 to 7.93)	⊕⊕○○ Low	No Studies were available from low-middle income countries. Studies reported a mix of population with varying baseline risk and surrogate outcome. Unexplained heterogeneity, downgraded one point each in inconsistency and indirectness in population
Cost-effectiveness of ezetimibe with statin therapy when compared to other lipid-lowering therapeutic agents/placebo in High-income countries (Assessed with meta-analysis).									
19	not serious	serious[a]	serious[d]	not serious	unlikely	4.36	(0.62 to 8.09)	⊕⊕○○ Low	Unexplained heterogeneity is due to difference in population and treatment groups included among HICs so not downgraded. Downgraded one point each in inconsistency and indirectness in population
Cost-effectiveness of ezetimibe with statin therapy when compared to other lipid-lowering therapeutic agents/placebo for primary prevention (Assessed with meta-analysis).									
6	not serious	not serious	serious[d]	serious[b]	unlikely	4.81	(2.52 to 7.11	⊕⊕○○ Low	No heterogeneity, downgraded one point from imprecision and indirectness.
Cost-effectiveness of ezetimibe with statin therapy when compared to other lipid-lowering therapeutic agents/placebo from a payers' perspective (Assessed with meta-analysis)									

Outcome: Cost-effectiveness (assessed with meta-analysis of cost utility analysis)									
**Quality assessment***						**Summary of findings**			**Comments**
**No of studies**	**Risk of Bias**	**Inconsistency**	**Indirectness**	**Imprecision**	**Publication Bias**	**Effect (*10^3 US$)**		**Certainty/Quality**	
						**INB**	**95%CI**		
4	not serious	serious[a]	serious[d]	serious[b]	unlikely	3.26	(0.57 to 5.94)	⊕◯◯◯ Very Low	Inconsistency is due to difference in population and treatment groups included. Unexplained heterogeneity downgraded one points each from inconsistency indirectness and imprecision. Only four studies included.
Cost-effectiveness of ezetimibe with statin therapy when compared to other lipid-lowering therapeutic agents/placebo for lifetime horizon (Assessed with meta-analysis)									
19	not serious	very serious[c]	not serious	serious[b]	unlikely	4.57	(0.75 to 8.40)	⊕◯◯◯ Very Low	Unexplained heterogeneity downgraded two points from inconsistency and one in imprecision.
Cost-effectiveness of ezetimibe with statin therapy when compared to other statin monotherapy for primary prevention (Assessed with meta-analysis)									
4	not serious	not serious	not serious	serious[b]	unlikely	4.99	(2.66 to 7.33)	⊕⊕⊕◯ Moderate	No heterogeneity downgraded one point each from imprecision. Only four studies included.
Cost-effectiveness of ezetimibe with statin therapy when compared to other statin monotherapy for payers' perspective (Assessed with meta-analysis)									
3	not serious	not serious	not serious	serious[b]	unlikely	2.03	(0.07 to 3.99)	⊕⊕⊕◯ Moderate	Low heterogeneity cannot be explained, downgraded one point from imprecision. Only three studies included.

. [a] inconsistency $I^2 \approx 100\%$ [b] studies included have reported a wide confidence intervals [c] high heterogeneity [d] Lack of generalisability *Since all included studies are model based Cost-Utility studies, we have not included the Study design and Number of participants under consideration for assessment.

GRADE Working Group grades of evidence
High certainty: we are very confident that the true effect lies close to that of the estimate of the effect.
Moderate certainty: we are moderately confident in the effect estimate; the true effect is likely to be close to the estimate of the effect, but there is a possibility that it is substantially different.
Low certainty: our confidence in the effect estimate is limited; the true effect may be substantially different from the estimate of the effect.
Very low certainty: we have very little confidence in the effect estimate; the true effect is likely to be substantially different from the estimate of effect
